# ERK3/MAPK6 promotes triple-negative breast cancer progression through collective migration and EMT plasticity

**DOI:** 10.3389/fonc.2025.1563969

**Published:** 2025-08-27

**Authors:** Sofia Morazzo, Soraia Fernandes, Marina Fortea, Helena Skálová, Daniel Pereira-Sousa, Marco Cassani, Kamila Vrzalová, Filip Kafka, Jan Vrbský, Mathilde Soulez, Sylvain Meloche, Ole Morten-Seternes, Veronika Bosáková, Jaeyoung Shin, Jan Frič, Kristina Haase, Giancarlo Forte

**Affiliations:** ^1^ International Clinical Research Center (ICRC), St Anne’s University Hospital, Brno, Czechia; ^2^ Department of Biology, Faculty of Medicine, Masaryk University, Brno, Czechia; ^3^ European Molecular Biology Laboratory, Barcelona, Spain; ^4^ Department of Biochemistry, Faculty of Science, Masaryk University, Brno, Czechia; ^5^ Institute for Research in Immunology and Cancer, Montreal, QC, Canada; ^6^ Department of Pharmacology and Physiology, Faculty of Medicine, Université de Montréal, Montreal, QC, Canada; ^7^ Department of Pharmacy, UIT The Arctic University of Norway, Tromso, Norway; ^8^ AtoGen Co., Ltd., Daejeon, Republic of Korea; ^9^ Institute of Hematology and Blood Transfusion, Prague, Czechia; ^10^ International Clinical Research Center (ICRC), Faculty of Medicine, Masaryk University, Brno, Czechia; ^11^ School of Cardiovascular and Metabolic Medicine and Sciences, King’s College, London, United Kingdom

**Keywords:** Triple-negative breast cancer (TNBC), Extracellular signal-regulated kinase 3 (ERK3), epithelial-to-mesenchymal transition (EMT), epithelial-mesenchymal plasticity (EMP), collective migration, mitogen-activated protein kinase 6 (MAPK6)

## Abstract

Triple-negative breast cancer (TNBC) is the most aggressive subtype of breast cancer, in which epithelial-to-mesenchymal transition (EMT) plasticity is required for successful metastasis. ERK3 has been implicated in promoting breast cancer migration and invasion, but the mechanisms remain elusive. Here, we investigated ERK3 expression across patient-derived datasets and explored its role in promoting EMT plasticity using different 2D and 3D *in vitro* models to investigate cell-extracellular matrix adhesion, migration and invasion, anchorage-independent growth, extravasation and colonization. We have established an association between ERK3 overexpression and aggressive breast cancer phenotypes, higher tumour plasticity, as informed by its grade, and poor clinical outcomes. Based on the hypothesis that ERK3 contributes to TNBC progression by supporting a partial-EMT state, we showed that ERK3 contributes to different steps of the metastatic process, especially by enabling collective migration but also by modulating other functional aspects related to an active EMT program. In conclusion, our results demonstrate that ERK3 contributes to TNBC progression and potentially metastasis by promoting EMT plasticity and collective migration.

## 1 Introduction

Triple-negative breast cancer (TNBC) is the most aggressive subtype of breast cancer. Chemotherapy remains the first—and largely the only—treatment available. However, TNBC presents a poor prognosis due to high recurrence and metastasis (1). These aspects are strongly correlated with the high cell plasticity of TNBC, which is associated with the emergence of cancer stem cells and features of epithelial-to-mesenchymal transition (EMT). EMT refers to the process by which cells transition from an epithelial (E) to a mesenchymal (M) state (2, 3). However, EMT can also involve intermediate states, referred to as hybrid E/M or partial-EMT, in which cells exhibit high EMT plasticity (2). A consensus from the EMT International Association defines hybrid E/M states as epithelial–mesenchymal plasticity (EMP) and characterises them by alterations in several molecular markers, such as E-cadherin, cytokeratins (KRT8, KRT18, KRT5, KRT14), SNAIL, and the Integrin Subunit Beta 3 ITGB3 (CD61), alongside morphological and behavioural changes. These include loss of apical–basal polarity, decreased cell–extracellular matrix (ECM) adhesion, or the gain of migratory and/or invasive capabilities (3, 4). In particular, EMP is required for breast cancer cells to metastasise by promoting collective migration through a basal program (2, 5). This basal program is characterised by KRT14 enrichment in leader cells and is strongly associated with high metastatic burden in *in vivo* models (5).

The mitogen-activated protein kinase (MAPK), such as the classical MAPKs—extracellular signal-regulated kinases 1/2 (ERK1/2), ERK5, p38 isoforms, and the c-Jun N-terminal kinase (JNK) isoforms—have been strongly implicated in cancer progression, including breast cancer and EMT (6–9). ERK3, encoded by the *MAPK6* gene, is considered an atypical MAPK, in part due to its constitutive activity in both physiological and pathological conditions, with a high turnover rate regulated by degradation, indicating that many functions are dependent on protein abundance (10–12). ERK3 has a protumourigenic role in several types of cancer, such as non-small cell lung cancer, and prostate, ovarian, cervical, and gastric cancers (11, 13–17). In breast cancer, ERK3 is essential for filopodia formation and modulates changes in cell body area, leading to more efficient cancer cell migration and invasion capacity (15, 18). Additionally, ERK3 has been shown to be involved in the formation of lung metastasis in *in vivo* models of TNBC (14, 16). However, the mechanism through which ERK3 leads to a high metastatic burden remains unclear.

In this study, we investigated the role of ERK3 in TNBC progression, focusing particularly on cancer cell migration and metastasis. First, we assessed ERK3 expression changes across breast cancer from patient-derived datasets, showing that its overexpression correlates with clinical parameters such as tumour subtype, tumour grading, and survival metrics. We then demonstrated, using four stable cell lines in which we silenced ERK3, that ERK3 contributes to EMP by (i) enhancing the expression of key molecular markers such as SNAIL and KRT14, (ii) promoting prometastatic pathways such as Wnt/β-catenin and yes-associated protein (YAP)/CYR61, and (iii) enhancing the collective migration of TNBC cells. Finally, we demonstrated the role of ERK3 in the metastatic capacity of TNBC cells using advanced 3D models, including a 3D microphysiological system (3D-MPS) to mimic the lung capillaries and a co-culture system of TNBC spheroids with human-induced pluripotent stem cell (hiPSC)-derived lung organoids.

## 2 Materials and methods

### 2.1 Patient-derived database analysis

Patient-derived datasets regarding ERK3/MAPK6 gene expression in normal breast tissue, breast invasive carcinoma, or metastatic breast invasive carcinoma were retrieved from the Gene Expression Omnibus of the National Center for Biotechnology Information (NCBI-GEO) (19), Genotype-Tissue Expression (GETx) (20), The Cancer Genome Atlas Pan-Cancer (TCGA) (21), and the Therapeutically Applicable Research to Generate Effective Treatments (TARGET) (22), all accessed through the Tumour, Normal and Metastatic tissue plotter (TNMplot; https://tnmplot.com/) (23). Also, ERK3/MAPK6 gene expression across the different subtypes of breast cancer and between different tumour grades, from the NCBI-GEO (19) datasets (Affymetrix U133A or U133Plus2), was accessed through the Gene Expression database of Normal and Tumour tissues platform (GENT2; http://gent2.appex.kr/gent2/) (24). Patient survival curves for either high or low ERK3/MAPK6 expression, including patient overall survival (OS), recurrence-free survival (RFS), and distant metastasis-free survival (DMFS), were accessed and analysed in the Kaplan–Meier plotter (https://kmplot.com/analysis/) (25), which derives survival data from several datasets deposited in the European Genome-phenome Archive (EGA), NCBI-GEO (19), and TCGA project (21).

### 2.2 Cell culture and reagents

Human immortalised breast cancer cell lines MDA-MB231 (CRM-HTB26™) and BT549 (HTB122™), lung cancer cell line A549 (CCL-185™), and pancreatic cancer cell line MIA PaCa-2 (CRL-1420™) were purchased from the American Type Culture Collection. MDA-MB231 and MIA PaCa2 were cultured in Dulbecco’s modified Eagle’s medium (DMEM) high-glucose (Biosera), supplemented with 10% fetal bovine serum (FBS), 1% penicillin–streptomycin, and 1% l-glutamine (all from Serana). BT549 and A549 were cultured in RPMI-1640 with l-glutamine, supplemented with 10% FBS and 1% penicillin–streptomycin. Human umbilical vein endothelial cells (HUVEC; C2519A) and normal human lung fibroblasts (nhLF; CC-2512) were purchased from Lonza and cultured in coated flasks with 50 μg/ml rat tail collagen I (Roche), using Vasculife (LL-0003) or Fibrolife (LL-0011), respectively, from Lifeline Cell Systems. All cell cultures were utilised until passage 10 or less to avoid genetic drifting and were routinely tested for mycoplasma contamination using MycoAlert™ Mycoplasma Detection Kit (Lonza) with a Centro LB960 luminometer (Berthold Technologies).

Human iPSC (WiCell, DF19-9-7T)-derived lung organoids (LO) were cultured, differentiated, and characterised as previously described (26–29). They were embedded in Cultrex reduced growth factor basement membrane, type 2 (~ 4 mg/ml, Cultrex, RGF BME, Type 2; R&D Systems) and maintained in LO complete media, which consists of LO basic media—Advanced DMEM/F12 media (Thermo Fisher Scientific), with HEPES (15 mM, Sigma), penicillin/streptavidin (500 U/ml), GlutaMAX (2.5 mM, Thermo Fisher Scientific), and N2 and B27 supplements (Thermo Fisher Scientific)—supplemented with HyClone™ FBS (1%, Thermo Fisher Scientific) and FGF-10 (500 ng/ml, R&D Systems) before each use. Media were changed twice a week, and LOs were used between 70 and 120 days in culture.

Standard-of-care drugs—doxorubicin (Sigma-Aldrich), carboplatin (Sigma-Aldrich), and paclitaxel CRS (European Directorate for the Quality of Medicines & HealthCare)—were used as a five-point twofold dilution curve. The cells were incubated for 48 h. Control cells were incubated with dimethyl sulfoxide (DMSO) in cases where the drug was dissolved in DMSO (doxorubicin and carboplatin). The synthesis of the small-molecule ERK3 inhibitor [3‐(4‐methoxy‐phenyl)‐3H‐[1,2,3] triazolo[4,5‐d]pyrimidin‐5‐yl]‐((R)‐1‐pyridin‐4‐yl‐pyrrolidin‐3‐yl)‐amine (referred to as ERK3i in the text) was performed as previously reported (30, 31). The compound was used at a final concentration of 10 μM, and control cells were incubated with an equivalent volume of DMSO for 20 h.

### 2.3 Induction of transient and stable knockdown

Transient knockdown of ERK3 was induced by small interfering RNA (siRNA) transfection using 20 nM of targeted siRNA (Silencer™ Select, assay ID: 142309) as well as 20 nM of nontargeting control (Silencer™ Select Negative Control), both purchased from Thermo Fisher Scientific, with SAINT-sRNA transfection reagent (SR-2003-01, Synvolux), according to the manufacturer’s protocol, under antibiotic-free conditions for 24 h. Assays were performed 48 h posttransfection. The efficiency of each transfection was evaluated by Western blotting, as described in Section 2.8.

Stable ERK3 knockdown cell lines were generated using lentivirally packaged short-hairpin RNA (shRNA) particles with a green fluorescent protein (GFP) tag and puromycin resistance (VectorBuilder). Lentiviral titration of three different targeting shRNAs was tested, paired with a scramble control, and the shRNA targeting sequence ATCCTTACATGAGCATATATT (MOI: eight viral particles/cell) was selected to generate stable cell lines based on knockdown efficiency and viability. Cells were transduced in antibiotic-free media with 8 µg/ml of polybrene (Santa Cruz) and selected with puromycin (1 µg/ml, Invivogen). A total of four different transduced cell lines were generated with shRNA targeting ERK3 (shERK3) and one with the scramble control (shWT). The efficiency of knockdown was evaluated by Western blot and real-time PCR (qPCR), as described in Sections 2.8 and 2.9, respectively.

### 2.4 Generation of ERK3-3xFLAG cell line

For the generation of the plasmid for inducing ERK3 overexpression, the full-length of wild-type ERK3 was amplified by PCR using Platinum Pfx polymerase (Invitrogen), pSG5-ERK3 (10) was used as a template, together with the primer pair ERK3 pSG TOPO forward (5′-CACCCGGAATTCGAATAGTAAGGGTTTCAAAATGGC-3′) and ERK3 3xFlag reverse (5′-GTCGACTCACTTGTCATCGTCATCCTTGATATCGATATCATGATCTTTATAATCACCGTCATGGTCTTTGTAGTCGTTCAGATGTTTCAGAATGC-3′). The generated PCR product (ERK3 3xFlag) was first TOPO-cloned into pENTR/D-TOPO, confirmed by sequencing, and subsequently recombined into pLenti-PGK-Blast-DEST (Addgene No. 19065) (32). Second-generation lentiviral packaging plasmids pMD2.g (#12259) and psPAX2 (#12260) were purchased from Addgene. These two plasmids and ERK3-3xFLAG were individually amplified in One Shot™ Stbl3™ Chemically Competent *E. coli* (C737303, Thermo Fisher Scientific), and DNA was isolated using a plasmid DNA isolation kit (Thermo Fisher Scientific). After, ERK3-3xFLAG was then validated by Sanger sequencing using the primers listed in **Table 1**.

**Table 1:** Primers used for Sanger sequencing.

Primer	Sequence 5′–3′
pMAPK6 Fw	GCATGCCAGGCTTTTCATGT
pMAPK6 Rv	TCAGTAGGCTCATGCTGGGA
hPGK Fw	GTGTTCCGCATTCTCAAG
attB2 Rv	TACAAGAAAGCTGGGTCGGC

For lentiviral production, HEK 293T cells (kindly provided by Dr. V. Pekarik, Department of Physiology, Masaryk University, Brno, Czech Republic) were transfected in antibiotic-free DMEM medium with 1,750 ng of pMD2.g, 3,250 ng of psPAX2, and 5,000 ng of ERK3-3xFLAG using Lipofectamine™ 3000 (Invitrogen), following the manufacturer’s protocol. After 24 h of incubation, the medium was changed. On the following day, the supernatant was collected, centrifuged at 2,000 × *g*, for 10 min at 4°C, filtered using a 0.45-µm filter, and stored at − 80°C. MDA-MB231 cells were transduced with a 1:5 proportion of lentiviral supernatant, in antibiotic-free media with 10 µg/ml of polybrene (Santa Cruz). A total of four different transduced cell lines were generated and validated by Western blot.

### 2.5 Migration and invasion assays

Transwell migration assay was performed using the colorimetric QCM Chemotaxis Cell Migration Assay (ECM508, Merck), in a 24-well plate format with 8 µm pore size. Following the manufacturer’s protocol, at 48 h post-siRNA transfection, 2.5 × 10^5^ cells/ml were plated in serum-free conditions (300 µl) in the top insert and with full media (300 µl supplemented with 10%FBS) in the bottom well. After 24 h, nonmigrating cells were gently removed using the cotton swab included in the kit. The remaining attached and migrating cells were stained using the kit’s extraction buffer and cell stain, then solubilised and transferred to a 96-well plate for spectrophotometric measurement at 560 nm using a Multiskan Go microplate reader (Thermo Fisher Scientific).

Wound healing assays were performed using either siRNA-transfected cells or shRNA-transduced stable cell lines in a six-well plate format. Wound area was created by scratching with a 200-µl tip when the cell culture reached approximately 70%–80% confluency. Two images of the wound area were acquired per well (two to three wells/condition) and per time point (0 h, 20 h), using a × 10 objective on a Leica DM IL Inverted Phase Contrast Microscope, and analysed using ImageJ software with the wound healing size tool plugin (33). To exclude potential contributions from cell proliferation, the proliferation rate was measured for each cell line used, as described in Section 2.17.

3D TNBC spheroids were generated using 2 × 10^5^ cells/ml with 0.05 mg/ml collagen I rat tail solution (Gibco A10483), plated in ultra-low attachment, round-bottom 96-well plates, and centrifuged at 1,000 rpm for 5 min, with five acceleration and two deceleration speeds. After 24 to 48 h, spheroids were completely formed and used for either invasion assays or confrontation assays. For the invasion assays, the spheroids were embedded in either approximately 4 mg/ml Cultrex (RGF BME, Type 2, R&D Systems) or 1 mg/ml collagen I solution, rat tail (Gibco A10483), in a four-well plate. Images were acquired using a Leica DM IL Inverted Phase Contrast Microscope and analysed using ImageJ software, with the manual selection tool, to measure the invading area at different time points (day 0, day 6). Their use in the confrontation assays is described in section 2.15.

### 2.6 Cell–ECM or Cell–dECM adhesion assays

Adhesion assay to different recombinant ECM proteins was performed using the colorimetric ECM Cell Adhesion Array Kit (ECM540, Merck), plating 3 × 10^4^ cells/well at 48 h post-siRNA-transfection. After 24 h of incubation, and following the manufacturer’s instructions, wells were washed three times with PBS (× 1). Using the kit’s extraction buffer and cell stain, the remaining attached cells were solubilised, extracted, stained, and measured in a 96-well plate by spectrophotometry at 560 nm using a Multiskan Go microplate reader (Thermo Fisher Scientific).

Decellularised ECM (dECM) adhesion assays were performed using either lung dECM hydrogel kit (CC175, Sigma-Aldrich) or liver dECM hydrogel kit (CC174, Sigma-Aldrich), according to the manufacturer’s instructions. Briefly, the hydrogels were prepared at 3 mg/ml and used to coat a 96-well plate suitable for fluorometric measurement (black, transparent bottom). Stable cell lines were plated at 3 × 10^4^ cells/well on top of each dECM. Additionally, to account for nonspecific adhesion effects, cells were cultured in similar conditions, in noncoated wells. After 24 h incubation, wells were washed three times with PBS, and the remaining cells were measured based on GFP fluorescence using a Biotek FLx800 Fluorescence Microplate Reader and quantified based on a standard curve.

### 2.7 Soft agar colony formation assay

Anchorage-independent growth was evaluated using the CytoSelect™ 96-Well Cell Transformation Assay (CBA-130, CellBioLabs), following the manufacturer’s instructions. Control and stable ERK3 KD cell lines were plated at 1.5 × 10^3^ cells/well. After 8 days in culture, cells were solubilised, lysed, and stained with CyQuant^®^ GR Dye, according to the manufacturer’s protocol, and measured by standard curve and fluorometric detection with the Biotek FLx800 Fluorescence Microplate Reader.

### 2.8 Western blot

Proteins were extracted from cell pellets using RIPA buffer (150 mM NaCl, 50 mM Tris, 1% Nonidet P-40, 0.5% sodium deoxycholate, pH 7.4), supplemented with 10 mM PMSF, protease inhibitor, and phosphatase inhibitor cocktails (1% v/v, P8340 and P5726, respectively, Sigma-Aldrich), and 1% SDS. Protein amount was determined using Pierce BCA Protein Assay Kit (Thermo Fisher Scientific), with albumin to generate a twofold dilution standard curve, and measurement performed by spectrophotometry (562 nm). Samples were then prepared with 8 µg total protein, 4 × Laemmli Sample Buffer (Bio-Rad), and 1 mM DTT, and denatured for 10 min at 95°C. They were loaded in 10% acrylamide gels (TGX Stain-Free™ FastCast™ Acrylamide Starter Kit, 10%, Bio-Rad), with a protein ladder (NZYColour Protein Marker II, NZYtech). Densitometric data were obtained by incubating with Clarity™ Western ECL Substrate (Bio-Rad) for 1 min, followed by acquisition using the ChemiDoc MP Imaging System (Bio-Rad) and ImageLab 6.0.1 software (Bio-Rad). Using stain-free technology, loading and transfer efficiency to the nitrocellulose membrane was visually evaluated. Densiometric data were normalised against anti-beta-tubulin (1:5,000, HRP-conjugated, ab21058, Abcam) or total protein obtained from stain-free images, which were generated from each blot utilised for protein expression determination. Whole membranes and corresponding stain-free blots are shown in the **Supplementary Material**.

Primary antibodies used included anti-ERK3 (1:500, mouse mAb M02, clone 4C11; Abnova) and anti-GFP (1:1,000, mouse mAb, sc-9996) from Santa Cruz Biotechnology. Antibodies purchased from Abcam included anticytokeratin 14 (1:500, ab181595 [EPR17350], rabbit) and anti-PRAK/MK5 (phospho T182, 1:500, rabbit, ab138668). Antibodies obtained from Cell Signaling Technology included anti-SNAIL (1:1,000, C15D3, rabbit mAb, No. 3879), anti-AKT (pan, 1:1,000, C67E7, rabbit mAb, 4691), anti-phospho-AKT (Ser473, 1:1,000, D9E XP^®^ rabbit, 4060), anti-MAPKAPK-5 (1:1,000, D70A10, rabbit mAb, 7419), anti-YAP (1:1,000, D8H1X XP^®^, rabbit mAb, No. 1474), anti-CYR61 (1:1,000, D4H5D XP^®^, rabbit mAb, No. 14479), anti-β-catenin (1:1,000, D10A8 XP^®^, rabbit mAb, No. 8480), anti-N-cadherin (1:1,000 (D4R1H XP^®^, rabbit mAb, 13116), and anti-DYKDDDDK Tag (1:1,000, D6W5B, rabbit mAb, 14793). The phospho-supervillin (P-SVIL, S245) and pan-supervillin (pan-SVIL) antibodies were generated as previously described (31) and were used at a 1:1,000 dilution. Briefly, the antibodies were produced by GenScript via rabbit immunisation with the synthetic phospho-peptide EVPR(pS)PEEEERRRVRC, which was coupled to KLH. Antibody purification was performed by affinity chromatography (31). HRP-conjugated anti-rabbit (1:2,000) and anti-mouse (1:2,000, Cell Signaling Technology) were used as secondary antibodies.

### 2.9 cDNA sample preparation and qPCR

RNA was extracted from the cell pellet, using High Pure RNA Isolation Kit (Roche), according to the manufacturer’s protocol, and cDNA was synthesised using Transcriptor First Strand cDNA Synthesis Kit (Roche), according to the manufacturer’s protocol. qPCR was performed using LightCycler 480 SYBR Green I Master (Roche) and StepOnePlus™ Real-Time PCR System (Thermo Fisher Scientific), according to the manufacturer’s protocol, with the primers listed in **Table 2**. Relative gene expression was determined by the delta–delta Ct method, using beta-actin (ACTB) as a reference gene.

**Table 2:** qPCR primers used for gene expression determination.

Gene name	Protein name	Forward primer 5′–3′	Reverse Primer 5′–3′
MAPK6	ERK3	GGTCTTGCACGGATCATGGA	GTGCACCTGCAAAAAGGGTT
YAP1	YAP	AGAAGAGGTACCATGCTGTCCCAGATGAACGTCACA	ACAACATCTAGAATCCCGGGAGAAGACACTGGATTT
CCN1	CYR61	CCCGTTTTGGTAGATTCTGG	GCTGGAATGCAACTTCGG
KRT14	Cytokeratin 14	TGAGCCGCATTCTGAACGAG	GATGACTGCGATCCAGAGGA
SNAI1	SNAIL	TCGGAAGCCTAACTACAGCGA	AGATGAGCATTGGCAGCGAG
CTNNB1	Beta-catenin 1	AAAGCGGCTGTTAGTCACTGG	CGAGTCATTGCATACTGTCCAT
ACTB	Beta-actin	TGACGTGGACATCCGCAAAG	CTGGAAGGTGGACAGCGAGG

### 2.10 RT profiler

Gene expression array was performed using the RT² Profiler™ PCR Array Human Wound Healing mRNA array (PAHS-121Z, QIAGEN), following the manufacturer’s instructions, on LightCycler Galaxy (Roche). Gene expression data were analysed using the GeneGlobe Analyzer from QIAGEN (https://geneglobe.qiagen.com/cz/analyze).

### 2.11 Device fabrication

For the extravasation assay, devices were fabricated as previously described (34) by mixing PDMS (SYLGARD™ 184 Silicone Elastomer Kit, Dow) with elastomer in a 10:1 ratio, following the manufacturer’s protocol. After mixing, the PDMS-elastomer solution was degassed, poured into the respective mould, degassed again, and cured at 60°C overnight. The mould conferred dimensions of 0.5 mm in height, 15 mm in length, and 3 mm in width for the central and media channels. Devices were then cut out, and biopsy punches of 1 and 2 mm diameter were used to create the entry to the central channel and the media ports, respectively. The devices were air-plasma bonded to disinfected glass slides and incubated again at 60°C overnight to restore their native hydrophobic state. Before seeding, devices were sterilised in a laminar flow hood under ultraviolet light for at least 30 min.

### 2.12 Microvasculature formation

Following the previously described protocol (34) to generate perfusable vasculature using the aforementioned device, fibrinogen derived from bovine plasma (F8630, Sigma) was prepared in advance at 12 mg/ml diluted in PBS. Before seeding, fibrinogen solution was filtered with a 0.2-µm pore-size filter, and thrombin (T4648, Sigma) stock solution (100 U/ml in 0.1% w/v bovine serum albumin solution) was diluted to a final concentration of 4 U/ml in cold VascuLife media. After trypsinisation with TrypLE, HUVECs (24 × 10^6^ cells/ml) and nhLF (4.8 × 10^6^ cells/ml) were individually mixed in the thrombin solution. Each cell–thrombin suspension was then combined in a 1:1 ratio with fibrinogen to reach a final concentration of 3 mg/ml. The cell–fibrinogen mixture (15–20 µl) was injected into the central channel of the device and incubated for approximately 20 min in a humidity chamber to allow the gel to polymerise. Subsequently, VascuLife media (150 µl) was added to the media ports, and devices were maintained in a large Petri dish (15 mm diameter) to minimise contamination, along with a smaller Petri dish (30 mm diameter) filled with PBS to avoid dehydration. The media were changed daily.

### 2.13 Extravasation assay

After 7 days in culture, cancer cells were injected into the devices. Immediately before adding the cancer cells, the devices were first incubated with 0.1% bovine serum albumin (BSA) in PBS, sterilised using a 0.2-µm pore size filter, for 15–20 min to prevent nonspecific adhesion, as previously described (35). Stable cell lines with and without ERK3 knockdown, labelled with GFP, were trypsinised using TrypLE and resuspended in VascuLife supplemented with 5% FBS. Approximately 4.6 × 10^4^ cells were injected through the media ports. The assays were performed under static conditions. Each biological replicate of shERK3, and different passages of shWT, were used in independent rounds (*N* = 3), consisting of six to eight devices per time point (6, 24, 48, and 72 h), with two devices per time point including controls (no injection of cancer cells, but all other steps performed identically). A total of three rounds were completed, one with each biological replicate. Following permeability measurements, the devices were washed with PBS and fixed with 4% paraformaldehyde (PFA) for at least 5 h, then washed three times with PBS and stored in 0.01% sodium azide in PBS, sealed with parafilm, at 4°C.

The devices were then stained with Wheat Germ Agglutinin, Alexa Fluor™ 647 Conjugate (WGA, Thermo Fisher Scientific) overnight at 4°C, and washed three times with PBS the following day before imaging. Images were acquired using a Zeiss LSM 780 confocal microscope. A central region was randomly selected, and using a × 20 objective with 0.6 zoom, the tilescan function (7 × 5 tiles) was used to acquire an area representing approximately 50% of the entire device. The z-stack function (3.6 µm step size) was used with a channel for GFP and T-PMT detection, and a channel for WGA 647. Images were then converted using Imaris File Converter 9.9.1 and processed in Imaris Viewer. Specifically, the background was subtracted, and images were aligned. Using the “surface” tool based on the WGA channel, the vessel network was determined. Subsequently, cancer cells were manually counted and classified in relation to the vessels. Specifically, if the entire cell body was within the vascular space, it was labelled as intravascular; if completely outside, as extravascular. In cases where the cell body was in both spaces, it was labelled as in mid-extravasation. Furthermore, many intravascular cells were observed in clusters, where possible, two or three cells were considered small clusters, while four or more cells were considered large clusters.

### 2.14 Permeability measurement

At each time point (24, 48, and 72 h), permeability was measured by removing the media and adding 40 µl of 70 kDa FITC-conjugated dextran (46945, Sigma-Aldrich) prepared at 0.1 mg/ml in VascuLife to one media channel. After approximately 1 min to allow perfusion, 40 µl of the same solution was added to the opposite media channel to stop the flow. Time-lapse images were acquired using a Stellaris 8 confocal microscope and LAS X Leica software. Specifically, a × 10 objective was used, and four random central regions of the device were selected to acquire a z-stack time-lapse image (3 × 5 min interval), with a 5-µm step size. Permeability—*P*(cm/s)—was calculated as previously described (34), in ImageJ, using maximum projection images from the dextran channel at *t* = 0 to generate a binary mask and outline the vessel perimeter and extravascular tissue area.

### 2.15 Confrontation assay

Human iPSC-derived lung organoids (LO) were co-cultured with TNBC spheroids generated from shRNA stable cell lines. The LO was placed and embedded in Cultrex RGF BME type 2 (~ 4 mg/ml, R&D Systems), and three TNBC spheroids of either shWT or shERK3 were positioned around the LO in close proximity, in four-well plates, for each biological replicate (N=4). After approximately 40 min of incubation at 37°C to allow the Cultrex to gel, LO complete media was added. Images were obtained using a Zeiss LSM 780 confocal microscope, detecting GFP signal and T-PMT/Bright Field, with a × 10 objective, tile scan function, and z-stack (40 stacks), for each time point (days 1–6 or day 8, every 24 h). After 6 or 8 days in culture, the confrontation assays were washed in PBS and fixed with 4% PFA for 45 min, then washed three times in PBS and incubated in 15% sucrose with 0.03% eosin at 4°C overnight. The following day, samples were embedded in optimal cutting temperature compound (OCT) for cryosectioning.

Autofluorescence observed in our samples from the confrontation assay was first quenched by incubating with Trypan Blue for 1 min, as previously reported (36–38), ensuring the quenching of both autofluorescence and GFP expression from the stable cell lines. Subsequently, the samples were stained with WGA, Alexa Fluor™ 647 Conjugate (Thermo Fisher Scientific) for 10 min, following permeabilisation in 0.2% Triton for 10 min, blocking for 30 min in 2.5% BSA, and incubation with anti-GFP conjugated with Alexa Fluor™ 488 (1:300, sc-9996, Santa Cruz Biotechnology) for 1 h, then DAPI for 15 min. After washing with PBS, slides were mounted with Mowiol overnight before acquisition. Images were acquired using a Zeiss LSM 780 confocal microscope and processed in ImageJ software.

### 2.16 Focal adhesion assay

Stable cells of MDA-MB231 shWT and shERK3 were plated in μ-Slide 8 Well (80806, Ibidi) at low confluency. When cells reached ~ 40%–50% confluence, they were fixed with 4% PFA for 10 min, washed in PBS, three times, followed by permeabilisation with 0.2% Triton for 10 min. Subsequently, a blocking step was performed for 30 min in 2.5% BSA prior to incubation with antivinculin (1:400, V9131, Sigma-Aldrich) overnight at 4°C. The following day, the samples were washed in PBS, incubated for 1 h at room temperature with the secondary antibody antimouse Alexa Fluor™ 555 (SA000042, Thermo Fisher Scientific, 1:500), followed by DAPI staining for 15 min. Samples were preserved in PBS with 0.02% sodium azide. Images of 10–15 cells per well were acquired using a Zeiss LSM 780 confocal microscope and processed using CellProfiler to measure focal adhesions. The pipeline used can be found in the **Supplementary Materials**.

### 2.17 Proliferation and viability assays

Proliferation activity was measured in transiently siRNA-transfected cell lines using PrestoBlue™ Cell Viability Reagent (Invitrogen), following the manufacturer’s protocol. This reagent was also used to measure viability in the chemoresistance assays. The proliferation of the stable cell lines was evaluated using Click-iT™ Plus EdU Alexa Fluor™ 647 Flow Cytometry Assay Kit (Thermo Fisher Scientific), according to the manufacturer’s protocol, in a Flow Cytometer (FACS Canto II, BD Biosciences), and then analysed using FlowJo software, version 10.

### 2.18 Statistical analysis

Experiments were performed using either independent siRNA transfections or four different stable cell lines generated by shRNA transduction via lentiviral delivery, to ensure reproducibility.

GraphPad Prism 10 was used to perform statistical analysis. Data are presented as mean ± standard deviation (SD). Statistical significance was evaluated using Student’s *t*-test, one-way ANOVA followed by Tukey’s multiple comparisons test, two-way ANOVA followed by Sídák’s multiple comparisons test, or a mixed-effects model followed by Tukey’s multiple comparisons test, as detailed in each figure caption. Data normality assessments were performed using the Shapiro–Wilk test for assays with small sample size (*N* = 3–6), while for data obtained from large datasets (*N* > 6), normality was evaluated using the D’Agostino and Pearson test. In the assays that did not pass the normality assessment, the nonparametric Mann–Whitney (Wilcoxon matched-pairs signed rank) test or the Kruskal–Wallis test were used, followed by Dunn’s multiple comparisons test. Survival curve analysis using the log-rank test was performed on the Kaplan–Meier plotter platform (25).

## 3 Results

### 3.1 ERK3 is mostly expressed in aggressive subtypes of breast cancer and correlates with poor clinical outcomes in patients

The significance of ERK3 in breast cancer was initially evaluated by exploring large multiomics datasets, including GETx (20), TCGA (21), and TARGET (22), as well as datasets from NCBI-GEO (19), through user-friendly platforms such as TNMplot (23), GENT2 (24), and the Kaplan–Meier plotter (25). ERK3 expression across patient samples was correlated with various clinical parameters.

**Figure 1:**
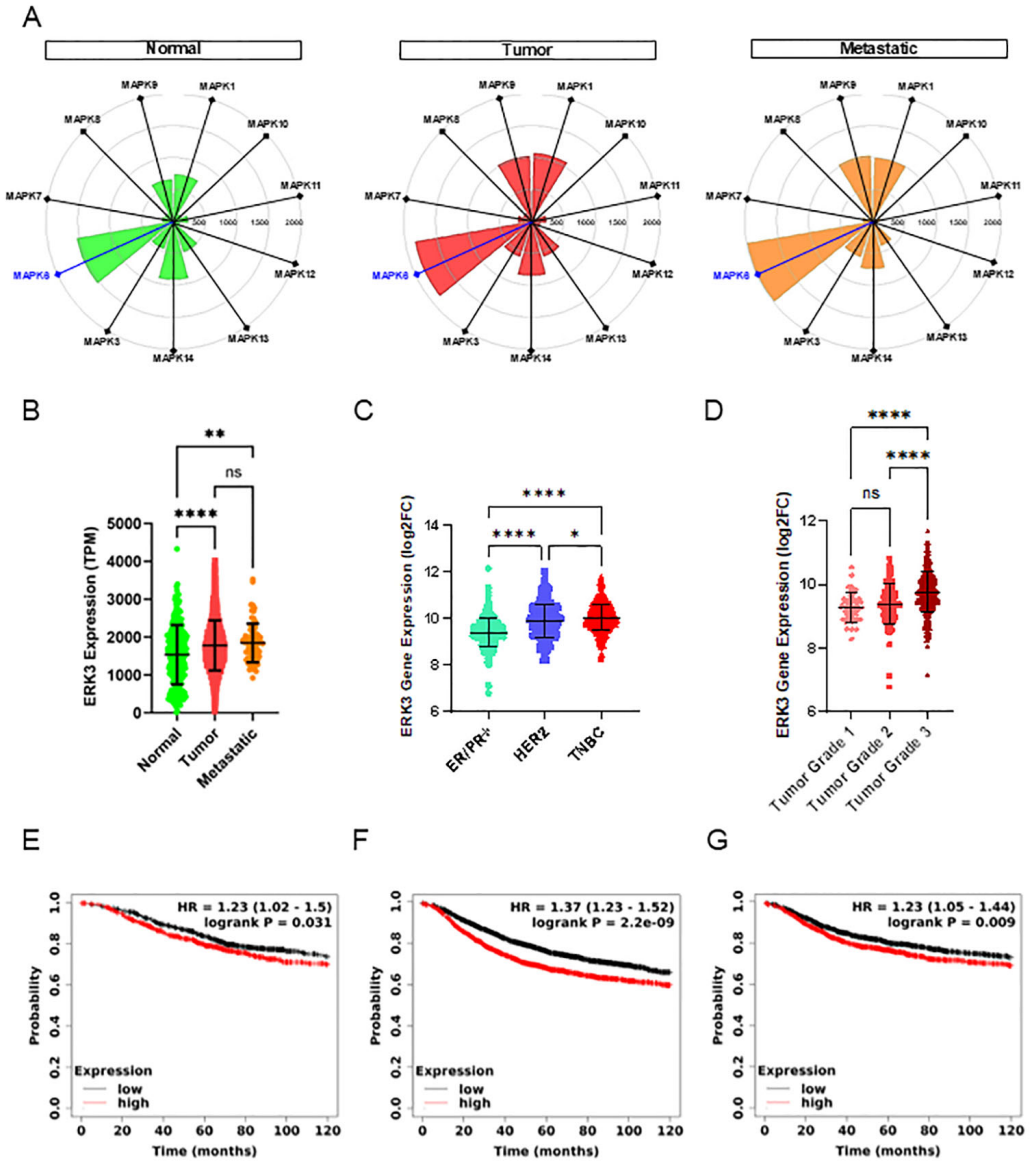
ERK3 overexpression in breast cancer patients correlates with poor patient survival. **(A)** Targetgram analysis of the average expression (transcripts per million [TPM]) of the different MAPK genes in normal breast tissue and primary and metastatic breast tumours, extracted directly from TNMplot (https://https://tnmplot.com/) (23). **(B)** Dot plot representation of ERK3 gene expression (TPM) in normal breast tissue and primary and metastatic breast tumours (normal *N* = 242, tumour *N* = 7440, metastatic *N* = 77), from the TNMplot. ERK3 gene expression is shown as log2 fold change (log2FC) from the GENT2 platform (http://gent2.appex.kr/gent2/) (24): distribution **(C)** by molecular subtype (ER/PR+ *N* = 623, HER2+ *N* = 229, TNBC *N* = 251), and **(D)** by breast cancer histological grade (grade 1 N = 63, grade 2 N = 151, grade 3 N = 358). Survival curves showing the probability of **(E)** overall survival (OS), **(F)** recurrence-free survival (RFS) and **(G)** distant-metastasis free survival (DMFS) in breast cancer patients patients with either high (red) or low (black) ERK3 expression. Data were accessed and analysed through the Kaplan–Meier plotter platform (https://kmplot.com/analysis/) (25). Dot plot graphs show data as mean ± SD. Statistical analyses were performed using the Kruskal–Wallis test followed by Dunn’s multiple comparisons test **(B**, **C)**; one-way ANOVA followed by Tukey’s multiple comparisons test **(D)**; and log-rank test. HR, hazard ratio **(E–G)**. ^*^ *p* < 0.05; ^**^ *p* < 0.01; ^****^ *p* < 0.0001; ns, nonsignificant.

First, we observed that ERK3, encoded by MAPK6, is significantly more expressed in normal breast tissue samples compared to other members of the MAPK family, such as ERK1/2 (MAPK3/MAPK1), ERK5 (MAPK7), the p38 isoforms (MAPK11, MAPK12, MAPK13, MAPK14), or the JNK isoforms (MAPK8, MAPK9, MAPK10, **Figure 1A**). Moreover, we found that ERK3 is overexpressed in tumours and metastatic breast cancer samples when compared to normal breast tissue (**Figures 1A, B**). Since interpatient variability can affect gene expression, we then verified ERK3 overexpression in primary tumours by analysing paired samples with normal-adjacent tissue from the same patient (**Supplementary Figure S1**). Second, given the significant impact of the distinct molecular subtypes of breast cancer on metastatic rate and prognosis, ERK3 expression was further examined based on the three established clinical groups: oestrogen/progesterone receptor positive (ER/PR+), human epidermal growth factor receptor 2 positive (HER2+), and TNBC (ER/PR/HER2−) subtypes (39). The analysis revealed that ERK3 is significantly more expressed in the most aggressive subtypes, i.e., HER2+ and TNBC (**Figure 1C**). Third, a key histopathological feature of TNBC, in comparison to the other subtypes, is the higher tumour grading (grade 3), which refers to less differentiated cells, higher plasticity, and more aggressive metastatic potential (40). The tumour grading system involves the pathohistological evaluation of tumours based on glandular differentiation, nuclear pleomorphism, and mitotic counts, ranging from grade 1 (well-differentiated glands, small and regular uniform cells, low mitotic count) to grade 3 (little to no glandular differentiation, high nuclear pleomorphism with over double nuclei size, vesicular chromatin, and high mitotic count compared to benign epithelial cells) (41). Our analysis shows that ERK3 expression correlates with tumour grading, being mostly expressed in grade 3 samples compared to grade 1 or 2 tumours (**Figure 1D**).

Finally, TNBC has the worst prognosis among breast cancer patients due to a higher recurrence rate, particularly within 5 years of diagnosis (42). Additionally, TNBC has a higher metastatic rate and, according to the Surveillance, Epidemiology, and End Results (SEER) program, the 5-year survival rate for localised TNBC drops from an average of 80% to 12% when distant metastases occur (43). Therefore, we analysed the impact of ERK3 overexpression in breast cancer patients, not only in OS, but also in RFS and DMFS. Our results indicate that high expression of ERK3 correlates with decreased OS (**Figure 1E**, 115 months for low expression vs. 81.6 months for high expression, HR = 1.23), RFS (**Figure 1F**, 64.9 months for low expression vs. 38.4 months for high expression, HR = 1.37), and DMFS (**Figure 1G**, 100 months for low expression vs. 68.4 months for high expression, HR = 1.23). Overall, our findings indicate that ERK3 expression correlates with a more aggressive breast cancer phenotype, as evidenced by increased expression in metastatic samples, higher tumour grade, and poorer survival outcomes in breast cancer patients, regardless of subtype, although more commonly associated with TNBC.

### 3.2 ERK3 promotes collective breast cancer migration and invasion

**Figure 2:**
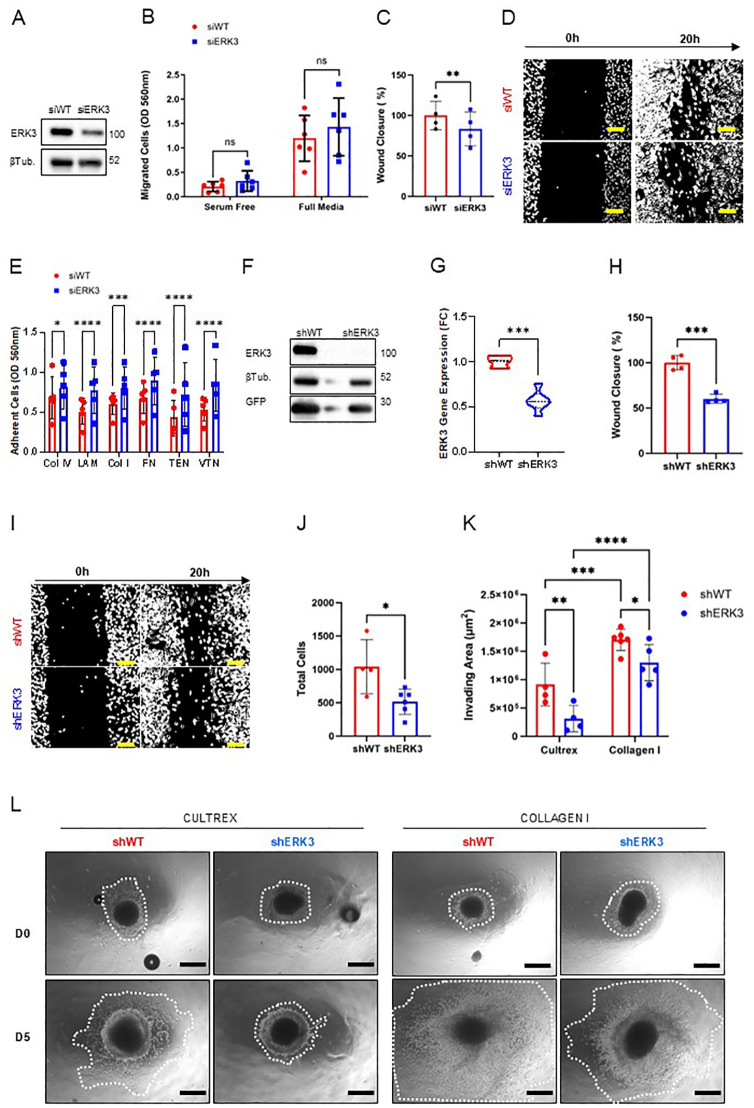
ERK3 promotes collective migration and invasion of TNBC cells. **(A)** Transient ERK3 knockdown in the MDA-MB231 cell line by siRNA and respective scramble control (siERK3 and siWT, respectively), validated by Western blot for ERK3 and β-tubulin (βTub). **(B)** Transwell migration assay of siERK3 or siWT cells seeded and incubated in the top insert for 20 h under serum-free or full media conditions in the lower compartment. Nonmigrating cells were scraped from the top insert membrane, while migrating cells were detached from the bottom of the insert membrane chamber and stained for colorimetric detection. Data are presented as mean ± SD. Statistical analysis was performed by two-way ANOVA followed by Šídák’s multiple comparisons test (*N* = 6). **(C)** Wound healing assay using siERK3 and siWT cells, measured after 20 h after the scratch; and **(D)** respective representative images of the wound healing assay at 0 and 20 h (scale bar: 100 µm). Data are shown as mean ± SD of the percentage relative to the shWT average wound closure. Statistical analysis was performed using a paired Student’s *t*-test (*N* = 4). **(E)** MDA-MB231 siERK3 and siWT cells were seeded on different ECM substrates for 20 h, after which nonadherent cells were washed off and adherent cells were quantified using a colorimetric method (Col IV, collagen type IV; LAM, laminin; Col I, collagen type I; FN, fibronectin; TEN, tenascin; VTN, vitronectin). Data are presented as mean ± SD. Statistical analysis was performed using two-way ANOVA followed by Šídák’s multiple comparisons test (*N* = 5). Stable MDA-MB231 cell lines with and without shRNA-induced ERK3 silencing (shERK3 and shWT, respectively) were validated by **(F)** Western blot for ERK3 and GFP, with β-tubulin (βTub) detection, and by **(G)** qPCR for ERK3 gene expression. Gene expression was normalised to beta-actin and shown as fold change (FC), using the delta–delta CT method. Data are presented as mean ± SD. Statistical analysis was performed using an unpaired Student’s *t*-test (*N* = 3). **(H)** Wound healing assay measured after 20 h after scratch of MDA-MB231 shERK3 and shWT stable cell lines; and **(I)** respective representative images (scale bar: 100 µm). Data are shown as mean ± SD of the percentage relative to the shWT average wound closure. Statistical analysis was performed by unpaired Student’s *t*-test (*N* = 4). **(J)** MDA-MB231 shWT and shERK3 cells were cultivated in a soft agar colony assay. After 8 days in culture, cells were solubilised, lysed, stained with CyQuant^®^ GR Dye, and measured by fluorometric detection. Data are presented as mean ± SD of total cell number, as determined by the standard curve. Statistical analysis was performed using an unpaired Student’s *t*-test (*N* ≥ 6). **(K)** Migration and invasion ability of MDA-MB231 shWT and shERK3 spheroids at days 0 and 5 of culture in either Cultrex or collagen. Data are shown as mean ± SD of the invading area. **(L)** Representative images of the invasion area (white dotted lines; scale bar: 250 µm). Statistical analysis was performed by two-way ANOVA followed by Šídák’s multiple comparisons test (Cultrex *N* = 4; collagen I *N* = 6). ^*^ *p* < 0.05; ^**^ *p <*0.01; ^***^ *p* < 0.001; ^****^ *p* < 0.0001; ns, nonsignificant.

We next set out to investigate whether ERK3 might play a role in collective and single-cell breast cancer migration, where the former is an indicator of EMP and is associated with more aggressive and efficient metastasis of breast cancer, while the latter is an indicator of full EMT and is associated with cancer recurrence (2). We transfected MDA-MB231 and BT549, two of the most aggressive TNBC cell lines (44), with siRNA against ERK3 or a scramble control (siERK3 and siWT, respectively, **Figure 2A**, **Supplementary Figure S2A**). Subsequently, we investigated the ERK3 effect on single-cell and collective migration using the Transwell and wound healing assays, respectively. For the Transwell migration assay, a serum-free condition was used as a negative control for chemoattraction, compared to the full media condition (containing 10% FBS). The results showed no differences in cellular migration rates in either MDA-MB231 or BT549, indicating that ERK3 does not contribute to single-cell migration in a general chemoattractant setting in breast cancer (**Figure 2B**, **Supplementary Figure S2B**). However, when investigating collective migration using the wound healing assay, we observed that ERK3 knockdown resulted in slower collective migration at 20 h in both TNBC cell lines (MDA-MB231 siERK3: − 16.62% ± 5.451%, compared to average siWT, **Figures 2C, D**, and BT549 siERK3: − 21.95% ± 4.445%, compared to average siWT, **Supplementary Figures S2C, D**). Additionally, the effect of siERK3 on proliferation was evaluated to rule out interference with the wound healing assay, confirming that ERK3 does not impact the proliferation of either MDA-MB231 or BT549 (**Supplementary Figures S2E, F**, respectively).

As the migration process is intimately connected with the balance of cell–ECM adhesion, a cell–ECM adhesion assay was performed to further address this question (45). The data shown in **Figure 2E** revealed that ERK3 knockdown significantly increased the adhesion of MDA-MB231 to various ECM substrates typical of breast tissue, including basement membrane proteins such as collagen type IV and laminin, as well as interstitial proteins such as collagen type I, fibronectin, tenascin, and vitronectin (46).

To better investigate the role of ERK3 in different metastasis-related processes, we established stable GFP-expressing MDA-MB231 cell lines using shRNA directed against ERK3 (shERK3). To increase the reproducibility of our assays, four biological replicates were generated, along with a non-targeting control (shWT). The stable cell lines were validated by Western blot and qPCR to confirm the downregulation of ERK3 (**Figures 2F, G**, **Supplementary Figure S2G**). Furthermore, we also investigated the effect of ERK3 knockdown on the phosphorylation status of its two main substrates: MAPK-activated protein kinase 5 (MAPKAPK5 or MK5) and protein kinase B (PKB or AKT) (47). The results showed that targeting ERK3 does not affect total MK5 or AKT protein expression, but does result in decreased phosphorylation of MK5 at T185 and AKT in S473, in accordance with previous studies (**Supplementary Figures S2H, I**) (10, 16).

Furthermore, we corroborated the results obtained in the wound healing assay by assessing cell migration at 0 and 20 h using the shERK3 and shWT stable lines. The results confirmed that ERK3 affects collective migration, as shown by the slower wound closure detected for shERK3 cells in comparison to shWT (shERK3: − 39.95% ± 4.698% compared to average shWT, **Figures 2H, I**). To validate this finding, ERK3 overexpression was induced in shERK3 cell lines by introducing ERK3-3xFLAG via lentiviral packaging, which was validated by Western blot (N=4) (**Supplementary Figure S3A**). The wound healing assay showed that, at 20 h, the cells exhibiting ERK3 overexpression had a higher migration rate compared to shWT cell lines (+ 36.48% ± 10.77%, **Supplementary Figures S3B, C**). This result validates that ERK3 directly regulates collective migration in breast cancer. 

It is known that ERK3 has both kinase-dependent and kinase-independent roles (15, 18, 48, 49). In MDA-MB231, the role in migration has been associated with a kinase-dependent role, via activation of CDC42 and RAC, and also with kinase-independent roles, particularly in migration on top of collagen-coated wells. Both studies associated ERK3 with cytoskeletal regulation, enhancing cell migration (15, 18). To investigate whether the effect of ERK3 on migration in our model is kinase-dependent, we utilised a selective ERK3 inhibitor, compound 18, which was previously described (30). Since ERK3 is constitutively phosphorylated on serine 189, the effect of ERK3 inhibitor (ERK3i) was validated by monitoring the phosphorylation of one of its *bona fide* substrate, supervillin, through Western blot analysis (**Supplementary Figure S3D**) (31). Our results showed that ERK3 inhibition at 10 μM significantly reduced cell migration (− 22.44% ± 3.6, **Supplementary Figures S3E, F**). While the reduction in migration was not as pronounced as that observed with the knockdown (**Figures 2H, I**), these findings are consistent with previous studies reporting that ERK3’s role in migration is partially kinase-dependent (15, 49). Together, these results suggest that ERK3 induces a promigratory phenotype via different mechanisms and may exert this effect by regulating the adhesion of breast cancer cells to the ECM.

Another process associated with reduced cell–ECM adhesion and enhanced metastasis is anchorage-independent survival (50). We investigated this phenomenon in our stable cell lines using the soft agar colony formation assay and showed that ERK3 promotes cancer cells’ ability to survive ECM detachment (**Figure 2J**). Next, to confirm that the results obtained from the wound healing and soft agar colony formation assays were not due to differences in proliferation activity between shERK3 and shWT cells, we used the Edu incorporation assay and demonstrated that ERK3 does not impact the proliferation of these cells (**Supplementary Figure S4A**). This outcome strongly supports the hypothesis that ERK3 modulates collective migration and survival mechanisms, without interference from breast cancer cell proliferation.

To further reinforce our findings regarding migration, we used a 3D model in which TNBC spheroids, generated with our stable cells lines (shWT, shERK3), were embedded in either Cultrex or collagen type I hydrogels and studied the migration and invasion of the cells in the presence or absence of ERK3 expression. The Cultrex 3D scaffold mimics the basement membrane of normal tissue, which is tipically degraded by invasive breast cancer cells. During this process, the invasive cells come into contact with collagen type I, an interstitial ECM protein that is abundant in the stroma of breast tissue and is increased in invasive breast cancer tissue (18, 46). The results of our experiment showed that TNBC spheroids from shWT were able to migrate and invade through both substrates more efficiently than those from cells with shERK3, as can be seen from the measured invading areas (**Figure 2K**). Moreover, we also observed that the overall migration and invasion ability of shWT and shERK3 cells was influenced by the type of ECM substrate used for the experiment, with enhanced TNBC migration through collagen I compared to Cultrex (**Figures 2K, L**).

Another feature associated with high plasticity and a partial-EMT state is increased drug resistance (2). Therefore, we investigated how ERK3 might affect TNBC cell survival under standard-of-care chemotherapy. The St. Gallen International Consensus Guidelines recommend, for TNBC patients, the use of an anthracycline such as doxorubicin or cyclophosphamide paired with a taxane, such as paclitaxel. For TNBC stages II and III, pairing with carboplatin is sometimes recommended (51). Accordingly, we performed a simple viability assay of the cells exposed to increasing concentrations of doxorubicin, paclitaxel (6.25–100 nM), or carboplatin (12.5–200 µg/ml) for 48 h. Our results showed that shWT cells are more sensitive to doxorubicin, particularly at lower doses—namely, 12.5 and 25 nM. However, at higher doses, such as 100 nM, this effect was reversed, with shWT cells showing higher resistance than shERK3 cells (**Supplementary Figure S4B**). Furthermore, shWT cells showed higher sensitivity to lower doses of paclitaxel, specifically 6.25 and 12.5 nM, while this effect is lost at higher doses (**Supplementary Figure S4C**). Importantly, we observed an overall resistance of shWT cells to most of the concentrations tested of carboplatin (**Supplementary Figure S4D**). These results indicate that ERK3 induces different levels of drug sensitivity in a dose- and drug-dependent manner.

Altogether, these results show that ERK3 promotes EMP features associated with metastasis by supporting collective migration and enhancing survival and invasion capabilities of TNBC cells.

### 3.3 ERK3 upregulates the expression of key EMT and prometastatic markers in cancer cells

Reduced cell–ECM adhesion and collective migration are two functional features characteristic of cells undergoing partial-EMT or EMP (3). Since ERK3 decreases cell–ECM adhesion and promotes collective migration in TNBC cells (**Figure 2**), we set out to investigate whether ERK3 facilitates EMP program expression in these cells.

Key drivers of EMP in TNBC are the transcription factor SNAIL and β-catenin (52). Importantly, breast cancer, including TNBC, requires a basal program to promote collective migration and successful metastasis, in which there is an enrichment of cytokeratin 14 (KRT14), particularly in leader cells (5, 53).

**Figure 3:**
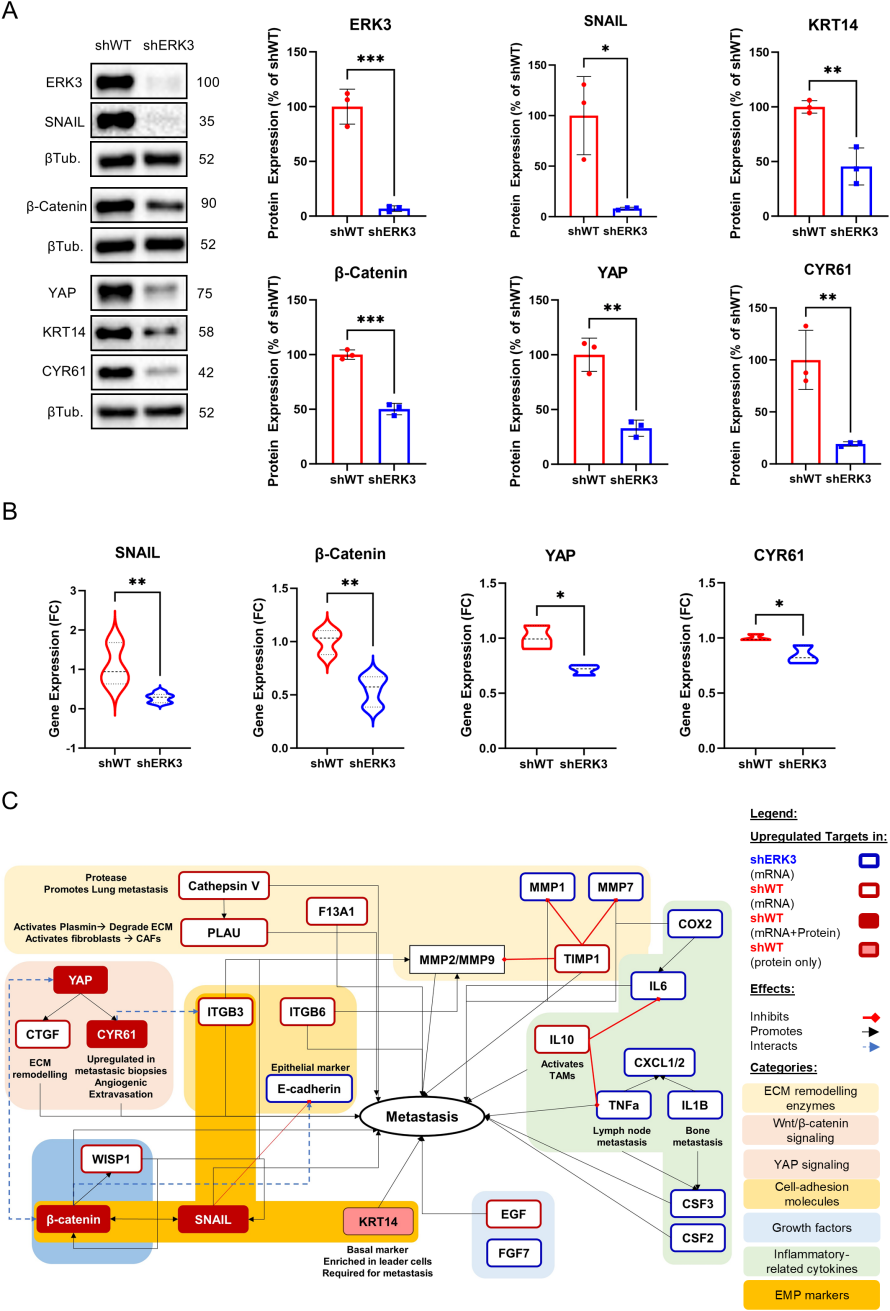
ERK3 upregulates EMT, prometastatic, and collective migration markers in TNBC cells. **(A)** Representative Western blot images and relative protein quantification of ERK3, SNAIL, β-catenin, YAP, CYR61, cytokeratin 14 (KRT14), and β-tubulin (βTub) in shERK3 and shWT stable cell lines. Data are presented as mean ± SD, shown as a percentage of the average protein expression of shWT cells after normalisation to β-tubulin. Statistical analysis was performed using an unpaired *t*-test. (*N* = 3). **(B)** Gene expression levels in shERK3 and shWT stable cell lines, determined by qPCR and expressed as fold change (FC) for SNAIL, β-catenin, YAP, and CYR61. Data are presented as mean ± SD, expressed as a percentage of the average FC of shWT cells after normalisation to β-actin, and calculated using the delta–delta Ct method. Statistical analysis was performed using an unpaired *t*-test. **(C)** Schematic representation of markers differentially regulated by ERK3, as identified by RT^2^ Profiler PCR Array (**Supplementary Table S1**), Western blot, and qPCR, and their association with the EMT and/or metastatic process in breast cancer, as reported in the literature. ^*^p < 0.05; ^**^p < 0.01; ^***^ *p* < 0.001.

Therefore, we evaluated the expression of the established EMP markers in breast cancer—SNAIL and KRT14—together with other key proteins involved in EMT, such as β-catenin, YAP and its downstream effector CYR61, in shERK3 and shWT cells. Western blot analysis, performed with our biological replicates (*N* = 3), showed that ERK3 silencing led to the downregulation of EMP markers SNAIL, β-catenin, and KRT14, as well as YAP and CYR61 (**Figure 3A**). Of interest, N-cadherin, which is a marker expressed in both partial- and full-EMT states, was not affected by ERK3 knockdown (**Supplementary Figure S4E**). A previous study in breast cancer showed that known EMT markers such as N-cadherin and vimentin are expressed similarly in both partial- and full-EMT states, while SNAIL expression is higher in partial-EMT states, with decreasing expression in full-EMT (48). According to these studies, our results suggest that ERK3 supports the partial-EMT state through upregulation of SNAIL.

Furthermore, to investigate if the observed downregulation of these markers also occurred at the transcriptional level, we performed qPCR, revealing that ERK3 silencing also reduces the transcription of the encoding genes for SNAIL (SNAI1), β-catenin (CTNNB1), YAP (YAP1), and CYR61 (CCN1) (**Figure 3B**).

To gain a better understanding of ERK3’s impact on the transcription of other important migration markers, we screened the expression, by RT-PCR Array, of 84 genes involved in cell migration and invasion (**Figure 3C**, **Supplementary Table S1**). Out of the 84 targets, 25 were differentially regulated in shERK3 and shWT cells. In shERK3 cells, we found the upregulation of 14 genes, which include several inflammatory cytokines (CSF3, IL1B, PTGS2, CXCL11, TNF, CXCL1, IL6, CSF2, and CXCL2), genes related to ECM degradation (MMP1, MMP7, and PLAT), the growth factor FGF7, and, notably, the gene encoding E-cadherin (CDH1), a key epithelial marker. In contrast, in shWT, there was the upregulation of 11 genes, 10 of which are known to promote metastasis in breast cancer. Among these genes, we found coagulation factor XIII (F13A1), which converts fibrinogen to fibrin, leading to the formation of clots and tumour emboli; the powerful anti-inflammatory cytokine IL-10; CCN family genes (CCN2/CTGF and CCN4/WISP1); integrins (ITGB3, ITGB6); ECM remodelling genes (CTSV, PLAU, TIMP1); the growth factor and EMT inducer EGF; and the tumour suppressor phosphatase and tensin homolog (PTEN) (**Figure 3C**, **Supplementary Table S1**) (50, 54–68).

Since partial-EMT is common in various cancer types, we further expanded our study to lung and pancreatic adenocarcinomas, given that they are among the most aggressive and mortality-inducing cancers (4). ERK3 is a well-established *bona fide* tumour-promoting gene in lung cancer, particularly in association with *PTEN* deletion and KRAS mutation, and its overexpression in patient samples correlates poorly with patient survival (11, 13). In contrast, ERK3 activity is less characterised in pancreatic cancer; however, literature reports that ERK3 overexpression also correlates with poor patient survival and promotes SNAIL stability (69). To investigate if ERK3 also modulates EMP in these types of cancer, we established stable cell lines of lung cancer A549 and pancreatic cancer MIA PaCa-2, with and without ERK3 knockdown, using the above-reported protocol, which were validated by Western blot (**Supplementary Figures S5A**, **S6A**). Subsequently, we investigated the effect on the EMP markers identified in MDA-MB231 in these cell lines. Our findings show that ERK3 regulates the expression of EMP markers in both lung and pancreatic cancer, in a cell-type-dependent manner. Particularly, ERK3 knockdown in pancreatic cancer has similar effects to those reported for breast cancer, resulting in reduced protein expression of YAP, KRT14, and SNAIL. However, it upregulated the protein expression of β-catenin and CYR61. Interestingly, ERK3 knockdown in lung cancer also downregulated YAP but upregulated β-catenin, CYR61, and SNAIL expression, while it had no significant effect on KRT14 (**Supplementary Figures S5A**, **S6A**). Next, we repeated the wound healing assay with the pancreatic and lung cancer cell lines. The results confirmed that shERK3 reduces collective migration in both cell lines (A549 shERK3: − 46.23% ± 5.84%, compared to average A549 shWT; MIA PaCa2 shERK3: − 24.22% ± 6.25%, compared to average MIA PaCa2-shWT; **Supplementary Figures S5B**, **S6B**, respectively). These findings suggest that ERK3 promotes collective migration in all the cancer types tested in this study, but possibly via different molecular mechanisms.

Overall, these findings show that ERK3 expression is linked to the expression of key partial-EMT markers in breast and pancreatic cancer—SNAIL, KRT14, and YAP—and it regulates the expression of protumour proteins such as β-catenin and CYR61 in a cancer type-dependent manner.

### 3.4 ERK3 increases cancer cell extravasation capacity

**Figure 4:**
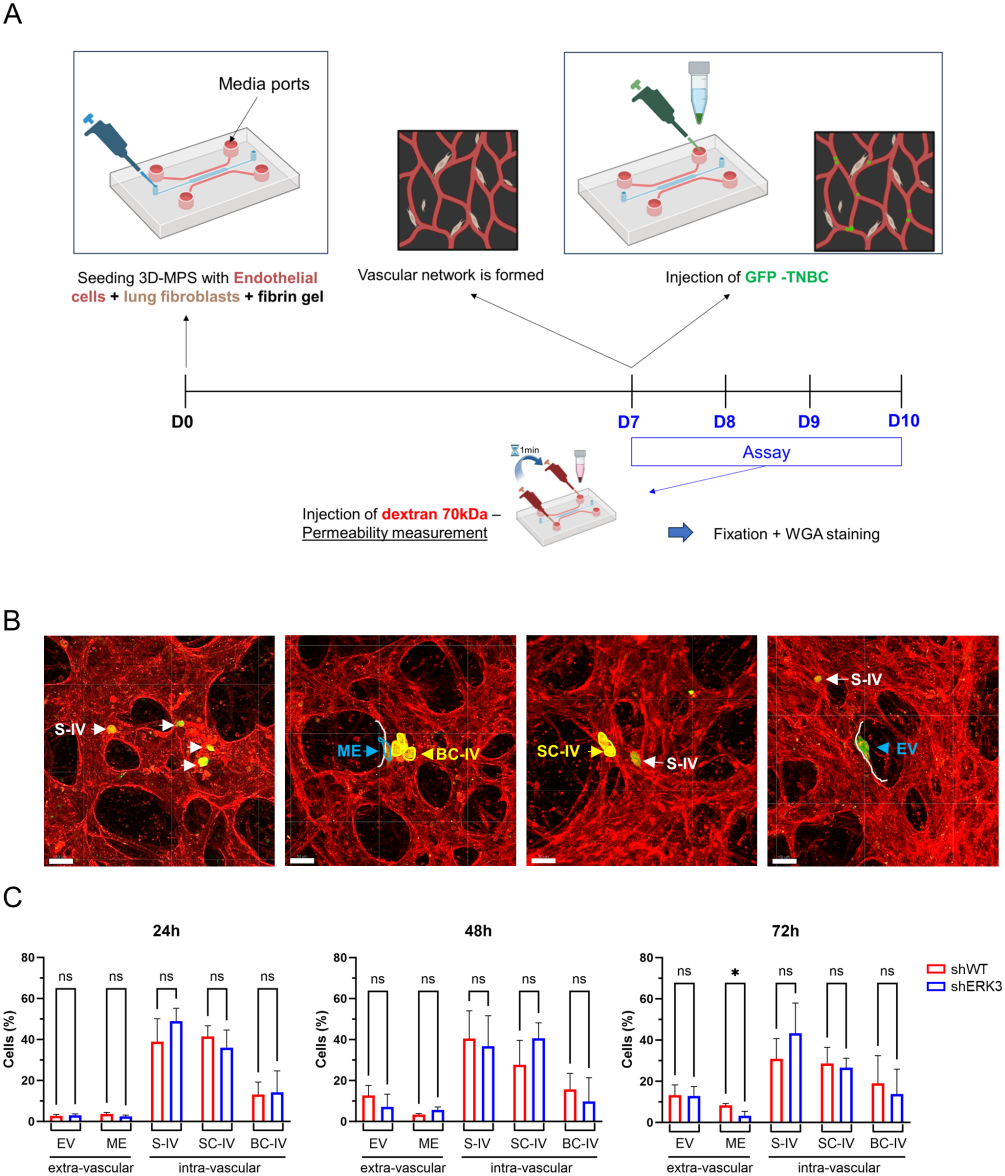
ERK3 increases cancer cell extravasation in a 3D microphysiological system that mimics lung capillaries. **(A)** Schematic representation of the 3D-MPS with a central channel (blue) used for seeding endothelial cells (red) and lung fibroblasts (brown) within a fibrin gel (top). The parallel media ports used for injection are indicated by an arrow. An experimental timeline of the assay is also shown (bottom). **(B)** Representative confocal images of vasculature stained with WGA (red), with GFP-labelled TNBC cells (green). Based on their position relative to the blood vessel wall (white line), TNBC cells were classified as intravascular (IV), extravascular (EV), or mid-extravasation (ME, blue). Additionally, depending on the number of cells found together, TNBC cells were further classified as single intravascular (S-IV, white) or clustered (yellow), specifically as small (two to three cells, SC-IV) or large (> 4 cells, BC-IV) intravascular clusters (scale bars: 50 µm) **(C)** Bar plot representation of TNBC cell classification as a percentage of total cells counted over time (24, 48, and 72 h). Data are presented as mean ± SD (*N* = 3). Statistical analysis was performed using two-way ANOVA followed by Sidak’s multiple comparisons test. ^*^p < 0.05; ns, nonsignificant.

Metastatic cancer cells are characterised by their ability to disseminate toward distant organs, where eventually secondary tumours are established (2). In this regard, TNBC cells have been shown to preferentially metastasise to the lung (1, 39, 40). In order to investigate the role of ERK3 in this process, we used a complex 3D-MPS system, in which lung fibroblasts and endothelial cells were seeded within a fibrin gel, creating a perfusable vasculature that mimics the normal human lung capillaries (**Figure 4A**). Using this setup, first, we investigated the permeability of the 3D-MPS (24, 48, and 72 h post-cancer cell injection) by adding a 70-kDa FITC-conjugated dextran solution to one media port, and after 1 min, adding the dextran solution to the opposite media port in order to stop the flow and acquire time-lapse images (**Supplementary Figure S7A**). No differences were detected in the diffusion of FITC-conjugated dextran between 3D-MPS inoculated with shWT or shERK3 cells, nor with a negative control (no injected cancer cells), and they were all within physiological parameters (**Supplementary Figure S7B**, approximately 10^−7^ cm/s), as previously reported (70). These results confirmed that the vasculature in our system was both permeable and perfusable. Subsequently, the same 3D-MPS were used to quantify TNBC cell extravasation at different time points (24, 48, and 72 h). In detail, the devices were immediately fixed after permeability measurement, and the cells were stained with WGA before confocal microscopy images were obtained and further processed using Imaris to improve the definition and visualisation of TNBC cells relative to the vascular network (**Figure 4A**). TNBC cells were classified as intravascular (IV—whole cell body is inside the vascular space) or extravascular (EV—whole cell body is outside the vascular space). Cells that were transmigrating to the extravascular space but with part of the cell body still visible in the IV space were classified as mid-extravasated (ME). Additionally, IV cells were subclassified into single IV cells (S-IV), small clusters (two to three cells, SC-IV), or large clusters (four or more, BC-IV) (**Figure 4B**). Quantification of the transmigration process showed an increase in mid-extravasated cells in shWT compared to shERK3 cells at 72 h (**Figure 4C**). This result supports the evidence of ERK3 playing a prometastatic role in TNBC.

### 3.5 ERK3 enhances the colonisation capacity of TNBC cells to lung organoids in *in vitro* models

**Figure 5:**
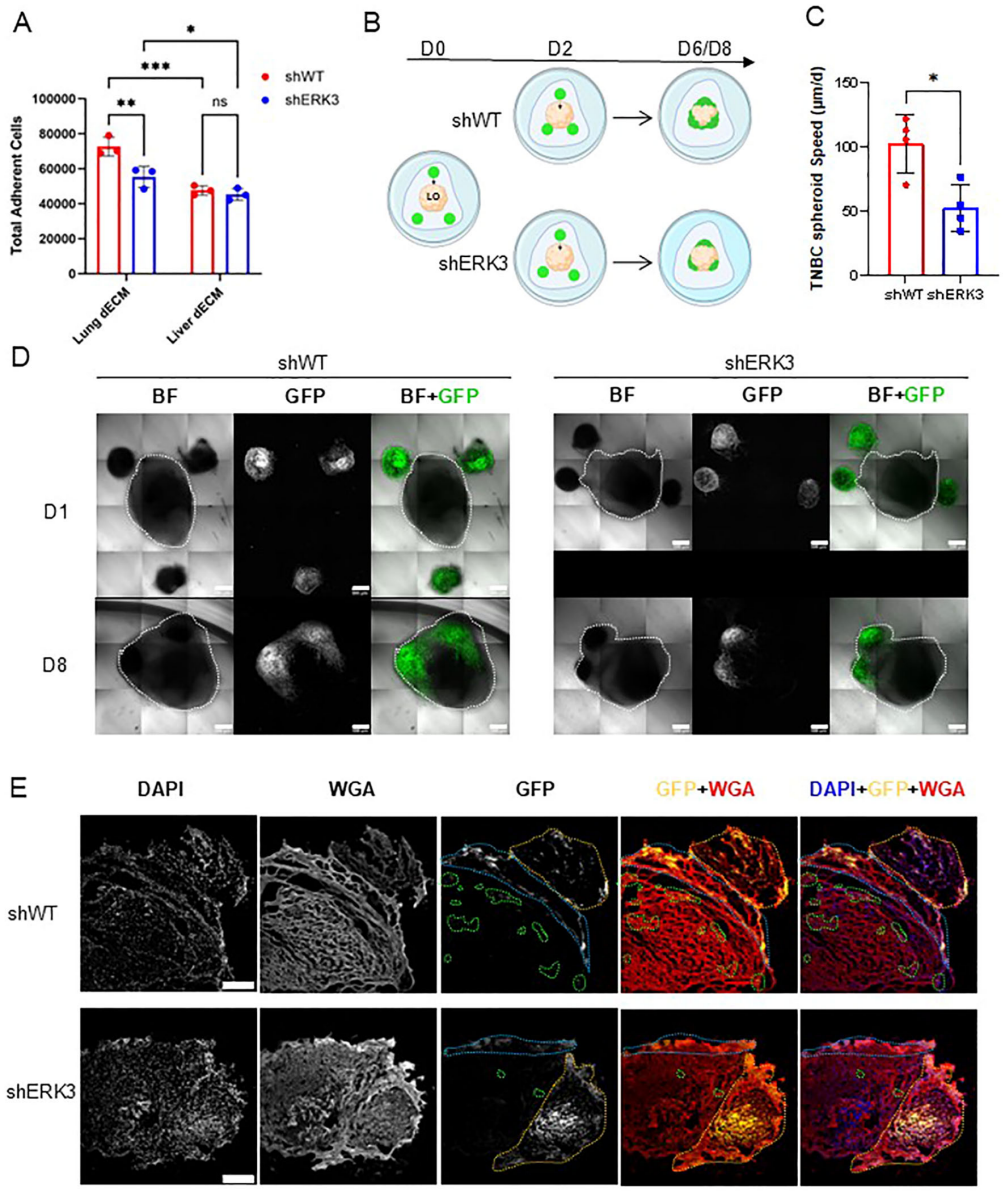
ERK3 promotes TBNC invasion into the lung in 3D *in vitro* models. **(A)** MDA-MB231 shERK3 and shWT cells were seeded on either lung dECM or liver dECM coatings for 20 h. Nonadherent cells were washed out, and adherent cells were measured by fluorometric detection. Data are presented as mean ± SD of total cells, determined using a standard curve. Statistical analysis was performed by two-way ANOVA followed by Šídák’s multiple comparisons test (*N* = 3). **(B)** Schematic of the confrontation assay, in which hiPSC-derived lung organoids (LO, beige) were co-cultured in Cultrex with the TNBC spheroids (in green) for a total of 6–8 days. **(C)** Average speed (µm/day) of TNBC spheroid migration toward the surface of the LO, estimated by the distances measured from confocal images acquired daily. Data are presented as mean ± SD. Statistical analysis was performed using an unpaired Student’s *t*-test (*N* = 4). **(D)** Representative images of the co-cultures obtained by confocal microscopy for shWT (left) and shERK3 (right) spheroids. Bright field (BF) and GFP-labelled cells at day 1 (D1) and after 8 days (D8) are shown (scale bar: 500 µm). The merge channel is also shown. **(E)** Representative confocal images of samples obtained from the confrontation assay, as in **(D)**, cultured for 8 days. The cryosections were stained with DAPI (blue), GFP (yellow) and WGA (red). The dashed orange line represents the spheroid, the blue line highlights the area of adhesion of the spheroid to the surface of the LO, and the green dashed line highlights penetrating cells or clusters of cells (scale bar: 250 µm). Data are presented as mean ± SD. ^*^p < 0.05; ^**^p < 0.01; ^***^p < 0.001.

In the final stages of the metastatic process, EMT plasticity confers breast cancer cells the ability to colonise the metastatic niche, as they retain the ability to undergo the reverse process called mesenchymal-to-epithelial transition (2). To further characterise ERK3’s role in confering EMT plasticity in favour of metastasis, we first investigated its effects on cellular adhesion using dECM from different organs, i.e., lung and liver. The adhesion assay revealed that, overall, cancer cells exhibit greater adhesion to lung compared to liver dECM, consistent with the overall preference for TNBC cells to colonise primarily the lung (**Figure 5A**) (39, 40). Interestingly, while no difference was observed between the two cell types regarding adhesion to liver dECM, shWT cells demonstrated increased adhesion to lung dECM compared to shERK3 cells. To validate these effects, an adhesion assay in noncoated wells was performed, and the formation of focal adhesions was assessed by vinculin staining and confocal microscopy measurements. No differences were found between shWT and shERK3 in their adhesion to noncoated wells (**Supplementary Figure S8A**), number of focal adhesions, focal adhesion area, or vinculin expression, as determined by mean fluorescence intensity. Furthermore, no changes were observed in cell area or cell eccentricity (**Supplementary Figures S8B, C**). These results are supported by previous reports showing that ERK3 modulates adhesion and migration in an ECM-sensitive manner (18), which differs from tissue to tissue.

In our previous experiments, we established that ERK3 participates in promoting migration and invasion in 3D models (**Figures 2H–L**) and adhesion to lung dECM (**Figure 5A**). To deepen our understanding of this process and contextualise it within the metastatic process, we investigated the colonisation capacity of TNBC spheroids in a 3D confrontation assay by co-culturing them with hiPSC-derived lung organoids (LO) (26). This methodology has previously been described to study the migration and invasion capacity of cancer cells (71). First, the GFP-tagged shWT or shERK3 TNBC spheroids were embedded in Cultrex in the proximity of the LO, and then the migration of the TNBC spheroids toward the LO was monitored for 6–8 days (**Figure 5B**). The approximate speed of the spheroid migration was extrapolated by calculating the distance between the surface of each TNBC spheroid and the surface of the LO, daily. Of note, TNBC spheroids placed at longer distances from the LO (approximately > 500 µm) did not migrate (respective spheroids are identified by blue numbers in **Supplementary Figures S9.1A**–**4A**, specifically shWT spheroids 3, 4, and 8; shERK3 spheroids 4 and 9). For all the other spheroids (distance < 500 µm, identified by yellow numbers in **Supplementary Figures S9.1A**–**4A**, specifically shWT spheroids 1, 2, 5, 6, 7, 9, 10, and 11; shERK3 spheroids 3, 5, 7, 10, and 11), the data displayed in **Figure 5C** shows that shWT spheroids moved faster than shERK3 spheroids (102.715 µm/day ± 22.463 µm/day vs. 52.884 µm/day ±18.832 µm/day, respectively). Furthermore, confocal live imaging revealed that, once in contact with the LO, the shWT spheroids adhered to the LO and the cells spread across a larger volume of the LO compared to the cells protruding from shERK3 spheroids (**Figure 5D**, **Supplementary Figures S9.1B**–**4B**). To corroborate this finding, on day 6 or 8 of culture, the co-cultures were fixed, cryosectioned, and stained with fluorescent markers for the cell membrane (WGA), GFP and the nucleus (DAPI). The results showed that the shWT spheroids infiltrated and colonised deeper into the LO, as depicted by the areas highlighted with orange, blue, and green dashed lines, while the cells from shERK3 spheroids remained at the peripheral sections of the LO (**Figure 5E**, **Supplementary Figures S9.1C**–**4C**). Of note, during the placement of the TNBC spheroids, five out of 12 shERK3 spheroids were placed in complete contact with the LO (respective spheroids identified by red numbers in **Supplementary Figures S9.1A**–**4A**, specifically shERK3 spheroids 1, 2, 6, 8, and 12), while no shWT spheroids were placed in such proximity. Nonetheless, the shERK3 spheroids presented a significantly lower invasion capacity in comparison to the shWT spheroids (**Supplementary Figures S9.1**–**4**).

These results highlight the interplay between ERK3 and the ECM in modulating cell adhesion, suggesting a reciprocal influence, and show that ERK3 enables efficient collective migration and invasion of TNBC cells toward normal lung organoids, a likely metastatic site for TNBC cells. Furthermore, our results align with previous reports using mouse models, in which injection of MDA-MB231 cell lines with or without ERK3 knockdown showed that ERK3 knockdown reduced overall tumour burden, cancer cell migration, and lung colonisation capacity (14, 15).

Altogether, these results support our hypothesis that ERK3 promotes increased TNBC cell plasticity and adhesion to secondary tissues, such as the lung.

## 4 Discussion

TNBC is the most aggressive subtype of breast cancer, characterised by high tumour grading and cell plasticity, resulting in increased recurrence and metastatic rate (39). Accumulating evidence implicates ERK3 signalling in breast cancer biology, with potential roles in tumour growth, migration, invasion, and survival (14–16, 18, 72, 73). Importantly, ERK3 has been shown to promote metastasis in *in vivo* models (11, 13–16, 74). Specifically, different studies using the TNBC cell lines MDA-MB231 or SUM159 have shown that ERK3 knockdown reduces metastatic burden due to decreased migration and invasion ability and reduced cancer cell survival (14–16). However, the specific role of ERK3 during cell migration and colonisation of secondary organs in TNBC remains unresolved.

Through the analysis of large multiomics datasets, we found that ERK3 has higher gene expression in normal breast tissue compared to the other MAPKs. Typically, ERK3 shows strong expression in glandular epithelia in breast samples (75). This might be due to ERK3’s high turnover and its key role in establishing epithelial architecture in mammary tissue by modulating mesenchymal-to-epithelial transition and cell–cell adhesion (10, 73). Furthermore, we showed that ERK3 is overexpressed in breast cancer patient samples, in both primary tumours and metastatic biopsies. Importantly, this increased expression correlates with aggressive subtypes of breast cancer, such as TNBC, higher tumour grading, and worse survival metrics (OS, RFS, DMFS). While we observed a correlation between increased ERK3 expression and the HER2+ subtype, these are very distinct types of breast cancer (HER2+ vs. TNBC), with distinct disturbances in molecular signalling, treatment responses, and overall cell behaviour (39). In this study, we investigated whether ERK3 may contribute to increased EMP and, consequently, to the metastatic process occurring in the TNBC subgroup.

Using MDA-MB231 as a model for TNBC, we observed that ERK3 promoted collective migration in a 2D model but had no influence on single-cell migration, which we also confirmed in another TNBC cell line, BT549. We further demonstrated that ERK3 promotes collective migration and invasion utilising a 3D spheroid model. Unlike conventional MAPKs such as ERK1/2, the effectors and signalling network of ERK3 remain poorly understood. Previous studies done in mammary epithelial and breast cancer cell lines have shown that ERK3 promotes cell migration by acting as a guanine exchange factor for CDC42 and Rac1 and by phosphorylating the ART3 subunit of the ARP2/3 complex, leading to filopodia formation at the leading edge and actin polymerisation (15). ERK3 was also reported to interact with septin 7 and diacylglycerol kinase eta, two known effectors of cell migration (48, 76, 77). Intriguingly, other studies have reported that ERK3 promotes migration in a kinase-independent fashion in both breast and lung cancers (15, 48, 49, 78). Furthermore, ERK3 was shown to regulate the cytoskeleton organisation and increase migratory speed in a kinase-independent but ECM-dependent manner (18). These results are in line with our observations that ERK3 modulates migration and cell adhesion in an ECM-dependent manner, and that the effect of ERK3 is, at least partially, kinase-dependent.

**Figure 6:**
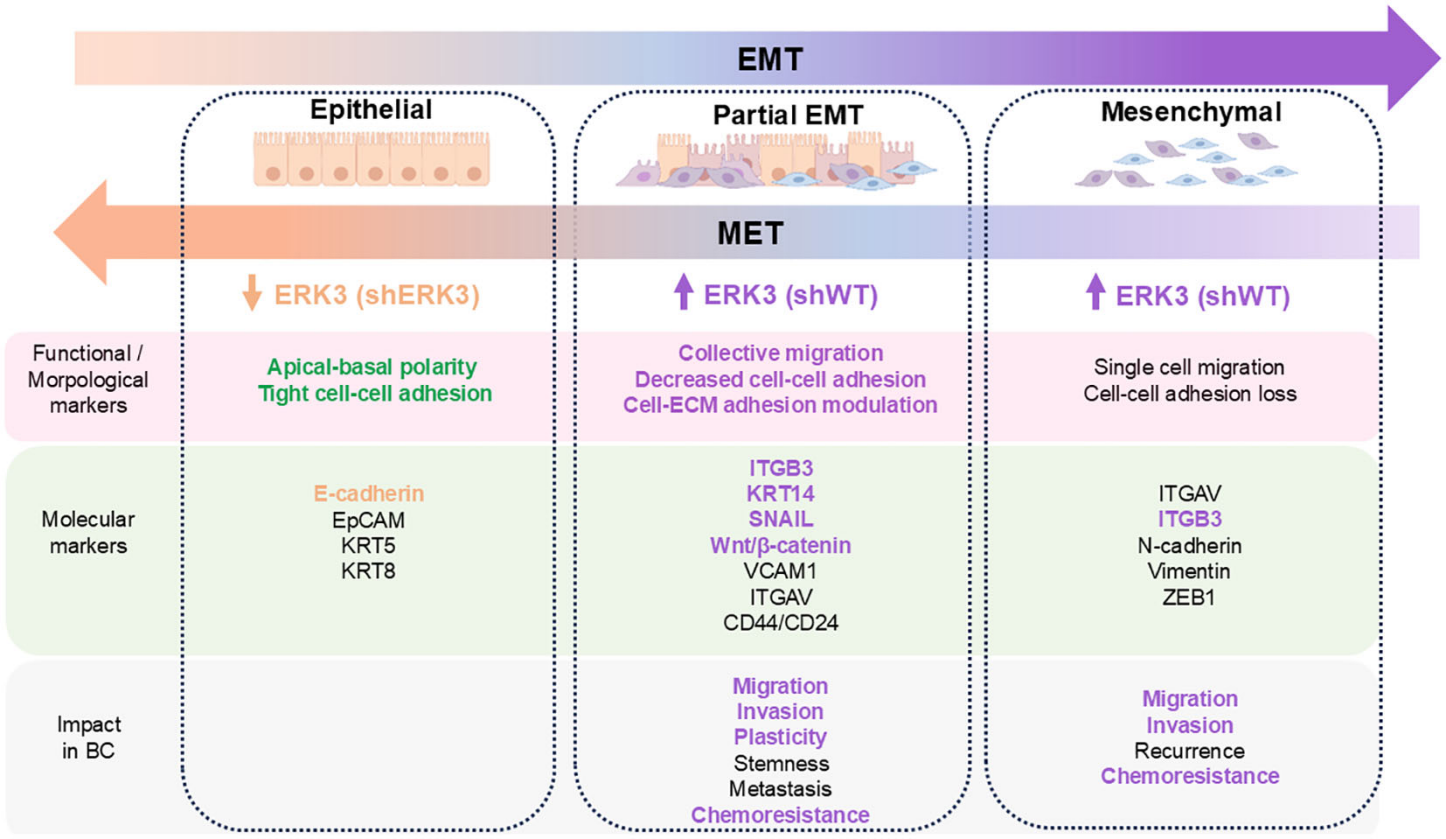
Summary of the different cellular states occurring during EMT, based on morphological, functional, and molecular markers, and their impact on breast cancer progression. The markers and impact of EMP and full EMT in breast cancer are listed based on recent evidence (3–5, 51, 52). Text is highlighted according to the characterisation of ERK3’s effect on these markers in our shWT (purple) and shERK3 cells (beige).

Collective migration is a key feature of a partial-EMT state, or EM plasticity (2–4, 53). Since EMT represents a spectrum of phenotypes rather than a binary switch, cells with higher plasticity often display intermediate stages of EMT, which should be defined by both changes in cellular behaviour and molecular markers (**Figure 6**) (3). Therefore, we investigated additional functional changes in cell behaviour characteristic of EM plasticity (EMP) and observed that ERK3 decreases cell adhesion to different ECM substrates, including collagen IV, laminin, collagen I, fibronectin, tenascin, and vitronectin, while promoting anchorage-independent survival. It has previously been reported that, upon its stabilisation by USP20, ERK3 decreases cell–ECM adhesion and promotes migration of various breast cancer cell lines (72). Recently, ERK3 has also been shown to phosphorylate AKT, promoting anchorage-independent survival in several types of cancer, including TNBC, through a mechanism associated with its role in increasing tumour burden in *in vivo* models (16). Our results strongly support the function of ERK3 in regulating these aspects of cell adhesion, survival, and migration, in accordance with the EMP phenotype.

Next, we investigated the expression of key molecular markers of EMP in TNBC. This intermediate state of EMT in breast cancer is mostly driven by the SNAIL transcription factor and canonical Wnt/β-catenin signalling (52). Additionally, KRT14 is known to be overexpressed in leader cells involved in the collective migration of TNBC and is also recognised as a marker of EMP (5, 53). Consistently, EMP leads to increased migration of invasive tumour cells through the breast tissue stroma (2, 5, 52, 53). In our study, we demonstrate that ERK3 knockdown reduces KRT14 protein expression and downregulates SNAIL and β-catenin at both transcriptional and protein levels in TNBC cells. Additionally, we demonstrate that the loss of ERK3 also downregulates YAP at both protein and transcriptional levels. YAP has been shown to interact with β-catenin in KRT14+ cells and to induce and sustain cancer stem cell survival, EMT, and overall TNBC progression (79). Moreover, we observed reduced gene expression of CDH1, which encodes E-cahderin, in shWT compared to shERK3, consistent with its typical repression by SNAIL. We also noted the downregulation of downstream effectors of β-catenin signalling and YAP, such as WISP1 (CCN4). CYR61 (CCN1), and CTGF (CCN2), all of which are implicated in promoting EMT (80, 81). Importantly, we also demonstrated that ERK3 appears to have a similar modulatory role in EMP in other types of cancer, specifically lung and pancreatic cancer, including a promigratory effect and regulation of SNAIL, KRT14, YAP, CYR61, and β-catenin. It has been reported that, upon stabilisation by Rab31 or SNHG19, ERK3 promotes EMT markers in cervical and lung cancers (82). Additionally, in pancreatic cancer, ERK3 protects SNAIL from degradation, thereby promoting SNAIL-driven EMT (69). Furthermore, other studies have shown that ERK3 can promote MMP expression, particularly MMP2, MMP9, and MMP10, linking this activity to its role in invasion (49, 78). Our results are consistent with these studies, showing that ERK3 also promotes EMT in breast cancer, and further that ERK3 regulates EMP-specific markers in these types of cancer.

After establishing the contribution of ERK3 to enhanced EMT plasticity through molecular and functional characterisations, we investigated whether this mechanism contributes to a higher metastatic capacity in TNBC cells. Using different advanced *in vitro* models—including organoids and microfluidic systems—we aimed to mimic the lung tissue, the preferred metastatic site of TNBC, and to study how cancer cells migrate toward and colonise this normal lung tissue model (39, 40). First, we examined the extravasation capacity using a 3D-MPS that mimics human lung capillaries. While we observed a lower extravasation rate compared to a previously reported study—which we attribute to the absence of constant flow in our system—our study revealed that ERK3 increases mid-extravasation of cancer cells after 72 h (35). As mentioned earlier, ERK3 upregulates CYR61 at both the protein and transcriptional levels. CYR61 has been shown to enhance breast cancer extravasation, possibly by promoting the survival of circulating tumour cells (83). In particular, CYR61 is overexpressed in relapsed TNBC tumours and metastatic biopsies (84). We also observed increased mRNA expression of one of CYR61’s receptors—ITGB3/CD61—which is a marker of late EMT stages and cancer stem cells (53, 84). Second, using decellularised ECM from either lung or liver, we demonstrated that ERK3 preferably promotes cellular adhesion to lung dECM, highlighting the interplay between ERK3-induced EMT plasticity and ECM specificity. Finally, we co-cultured human iPSC-derived lung organoids with TNBC cells, establishing an *in vitro* model of organ colonisation. This methodology was previously developed by our group to study the metastatic potential of patient-derived prostate cancer organoids toward lung organoids (71). Here, we used GFP-labelled tumour cells to track spheroid migration toward the lung organoid and to evaluate the penetration capacity of TNBC cells. Our observations showed that TNBC spheroids migrated cohesively toward the lung organoid, rather than radially, as observed in the 3D invasion assay using collagen I and Cultrex. Interestingly, using confocal microscopy and ImageJ to analyse the confrontation assay results, we demonstrated that ERK3 promotes faster spheroid migration toward the lung organoid, with approximately a twofold increase in average speed (µm/day) in shWT cells compared to shERK3 cells. Additionally, a qualitative analysis revealed deeper penetration of TNBC cells expressing ERK3 into the lung organoid, compared to those with ERK3 knockdown. These findings support the use of this model for future studies of cancer cell colonisation and, importantly, corroborate our hypothesis that the promigratory and proinvasive role of ERK3 contributes to TNBC metastasis, as previously reported in *in vivo* models (14–16).

In conclusion, our study shows that ERK3 promotes epithelial–mesenchymal plasticity in TNBC through the deregulation of several key molecular markers, including KRT14 and SNAIL, as well as important signalling pathways such as β-catenin, YAP, and CYR61. These alterations are supported by functional changes toward a partial-EMT state, characterised by the modulation of cell–ECM adhesion, anchorage-independent survival, collective migration and invasion, and differential responses to standard-of-care drugs. Using advanced 3D *in vitro* models, we corroborated the prometastatic behaviour of ERK3 in TNBC cells, particularly toward lung colonisation. The importance of our study is highlighted by the overexpression of ERK3 in more plastic and aggressive patient tumour samples and by its contribution to poor patient survival. Here, we were able to contribute to the understanding of how ERK3 promotes TNBC progression and possible metastasis formation, warranting future studies to determine whether ERK3 could serve as a valuable therapeutic target or biomarker in TNBC and other subtypes of breast cancer, such as HER2+.

## Data Availability

The original contributions presented in the study are included in the article/**Supplementary Material**. Further inquiries can be directed to the corresponding author.

## Supplementary material

Supplementary Table 1 Differentially regulated genes by ERK3, detected by RT² Profiler™ PCR Array Human Wound Healing (cut off values FC >1.5, P-value <0.05). The genes that passed the cut off threshold are highlighted in bold.

Supplementary Figure 1 ERK3 is overexpressed in primary tumour patient samples compared to normal tissue, adjacent to the tumour. Data, in transcripts per million (TPM), was extracted from the TNMplot database (tnmplot.com) and is shown as mean ± SD. Statistical analysis was performed with Wilcoxon matched pairs signed rank test. (N=70). *****P*<0.0001

Supplementary Figure 2
**(A)** ERK3 knockdown validation by western blot in BT549 cell line. siERK3 and siWT refer to cells transfected with siRNA targeting ERK3 and scramble control, respectively. **(B)** Transwell migration assay of BT549 siERK3 or siWT cells, for 20 hours, in serum-free or full media conditions. Non-migrating cells were scrapped from the membrane of the insert and the migrating cells were detached from the basolateral chamber and stained for colorimetric detection. Data is presented as mean ± SD. Statistical analysis was performed by two-way ANOVA followed by Šídák’s multiple comparisons test (N=6). **(C)** Wound healing assay using BT549 siERK3 and siWT cells, measured after 20 hours from the scratch. Data is shown as mean ± SD of the percentage relative to the shWT average wound closure, and Statistical analysis was performed by paired student *t-test* (N=4). **(D)** Representative images of the wound healing assay at 0 h and 20 h (Scale bar: 100 µm). Cell proliferation effect of siERK3 compared to siWT, measured with Presto Blue, in **(E)** MDA-MB231 and **(F)** BT549 cell lines, 72 hours post-transfection. Data is shown as mean ± SD of the percentage relative to the shWT average. Statistical analyses were performed by paired student *t-test* (N=6). **(G)** MDA-MB231 stable cell lines validation after lentiviral-mediated shRNA transduction of a scramble (shWT) or a shRNA targeting ERK3 (shERK3), by western blot for ERK3 and GFP, with β-tubulin (βTub.) detection. Representative images of western blot and relative protein quantification of **(H)** total and phosphoMK5 (P-MK5), and **(I)** total and phosphoAKT (P-AKT), in shERK3 and shWT stable cell lines. Data is shown as mean ± SD as a percentage of the average protein expression of shWT cells, after normalization with β-tubulin. Statistical analyses were performed by unpaired student *t-test* (H: shWT – N=5, shERK – N=10; I: N=3). ***P<0.01*, **P<0.05* or ns - non-significant.

Supplementary Figure 3
**(A)** Validation of four parallel lentiviral-mediated transductions of ERK3-3xFLAG, into shERK3 cells for ERK3 gain-of-function, by western blot for FLAG, ERK3 and GFP, with β-tubulin (β-tub.) detection. **(B)** Wound healing assay using shWT or shERK3 cells after transduction with ERK3-3xFLAG (shERK3+ERK3-3xFLAG), measured after 20 hours from the scratch. **(D)** Representative image of the western blot for validation of the ERK3 inhibitor, compound 18 (ERK3i), at 10 μM by its effect on decreasing phosphorylation of ERK3 substrate supervillin (P-SVIL), with no impact on total supervillin (SVIL), and detection of Hsc70. **(E)** Representative images of the wound healing assay using shWT, incubated with either DMSO or 10 μM of ERK3i, at 0 h and 20 h (Scale bar: 100 µm). **(F)** Wound healing area percentage measured 20 hours after the scratch. Data are shown as mean ± SD of the percentage relative to the shWT average wound closure. Statistical analyses were performed by unpaired student *t-test* (N=4). **P<0.05, ***P<0.001.*

Supplementary Figure 4
**(A)** ERK3 stable knockdown effect on proliferation of MDA-MB231 cells, measured by Edu-based proliferation assay, using flow cytometry. Data is shown as mean ± SD (N≥3). Statistical analysis was performed by unpaired student *t-test*. Cell viability after 48 hours incubation to a 2-fold dilution curve of either **(B)** doxorubicin (N=3), **(C)** paclitaxel (N=3), or **(D)** carboplatin, normalized to the cells incubated in DMSO (N=3). Data are shown as mean ± SD. Statistical analyses were performed with mixed-effects model, followed by Tukey’s multiple comparisons test. **(E)** Western blot of N-cadherin expression in shWT and shERK3 cells, with β-tubulin (βTub.) detection, and respective relative quantification of protein expression. Data is shown as mean ± SD as a percentage of the average protein expression of shWT cells, after normalization with β-tubulin. Statistical analysis was performed by unpaired student *t-test* (N=3). **P<0.05, **P<0.01*, *****P<0.0001 or* ns - non-significant.

Supplementary Figure 5
**(A)** Representative images of western blot and relative protein quantification of ERK3, SNAIL, β-catenin, YAP, CYR61, cytokeratin 14 (KRT14), and β-tubulin (βTub.) in A549 shERK3 and shWT stable cell lines. Data are shown as mean ± SD as a percentage of the average protein expression of shWT cells, after normalization with β-tubulin. Statistical analyses were performed with unpaired student *t-test* (N≥3). **(B)** Wound healing assay using A549 shERK3 and shWT stable cell lines, measured 20 hours after the scratch and respective representative image of the wound healing assay at 0 h and 20 h (Scale bar: 50 µm). Data is shown as mean ± SD of the percentage relative to the shWT average wound closure. Statistical analysis was performed by unpaired student *t-test* (N=3). **P<0.05, **P<0.01, ***P<0.001, ****P<0.0001* or ns - non-significant.

Supplementary Figure 6
**(A)** Representative images of western blot and relative protein quantification of ERK3, SNAIL, β-catenin, YAP, CYR61, cytokeratin 14 (KRT14), and β-tubulin (βTub.) in MIA PaCa-2 shERK3 and shWT stable cell lines. Data are shown as mean ± SD as a percentage of the average protein expression of shWT cells, after normalization with β-tubulin. Statistical analyses were performed with unpaired student *t-test* (N≥3). **(B)** Wound healing assay using MIA PaCa-2 shERK3 and shWT stable cell lines, measured 20 hours after the scratch and respective representative images of the wound healing assay at 0 h and 20 h (Scale bar: 50 µm). Data is shown as mean ± SD of the percentage relative to the shWT average wound closure (N=3). Statistical analysis was performed with unpaired student *t-test* (N=3). **P<0.05, **P<0.01, ***P<0.001.*

Supplementary Figure 7
**(A)** Representative confocal microscopy image of the whole 3D-MPS device, after 70 kDa dextran injection (red signal), with the four selected regions for time-lapse image acquisition indicated by dashed white lines. Scale bar: 500 µm. **(B)** Permeability measurement, in cm/s, of 3D-MPS devices, after 24-, 48- or 72-hours post shWT or shERK3 cancer cells injection. A negative control (CTL) was performed in the absence of cells injection. (24h: CTL N=3, n=5; shWT N=4, n=6; shERK3 N=4, n=7; 48h: CTL N=2, n=6; shWT N=3, n=5; shERK3 N=2, n=4; 72h: CTL N=2, n=4; shWT N=2, n=4; shERK3 N=2, n=5). Data shown as mean ± SD. Statistical analysis was performed with mixed-effects model, followed by Tukey’s multiple comparisons test. ns - non-significant.

Supplementary Figure 8
**(A)** MDA-MB231 shWT and shERK3 cells adhesion to non-coated wells after 20 hours. Non-adherent cells were washed out and adherent cells were measured by fluorometric detection. Data is presented as mean ± SD of total cells, determined by standard curve. Statistical analysis was performed by unpaired student t test (N=3). ns - non-significant. (**B**) Representative images acquired by confocal imaging, of shWT and shERK3 cells after staining for DAPI (blue), vinculin (red) and F-actin (purple), and detection of GFP (green). (Scale bar: 15 µm). (**C**) Focal adhesions and cell area quantifications, as detected by vinculin F-actin staining, respectively. Total focal adhesion per cell, focal adhesion area, mean intensity of vinculin expression per cell, number of focal adhesions per cell area, as well as total cell area and cell eccentricity measurements are shown. Data are presented as mean ± SD. Statistical analyses were performed by unpaired student *t-test* (n = 18 cells, N=3). ns - non-significant.

Supplementary Figure 9.1 Confrontation Assay – Biological Replicate 1 **(A)** Representative images obtained by confocal microscopy, using bright field (BF) and detection of GFP labelled cells at day 1 (D1) and after 8 days (D8) in culture for shWT (left) and shERK3 (right) spheroids. (Scale bar: 500 µm). **(B)** Representative confocal images of sectioned samples obtained from the same confrontation assay, cultured for 8 days. The cryosections were stained with DAPI (blue) and WGA (red). (Scale bar: 500 µm). **(C)** Representative images of the spheroids, highlighted by orange dashed line, the adhering area (dashed blue line) and penetrating cells (in dashed green line). (Scale bar: 250 µm). Spheroids are numbered across the biological replicates, in shWT and shERK3, from 1 to 11 or 1 to 12, respectively. Based on observation, they are colour coded in migrating spheroids (yellow), non-migrating spheroids (blue), or spheroids placed in direct contact with the LO (red). Same spheroids identified in panel A, are identified respectively in panel B and C.

Supplementary Figure 9.2 Confrontation Assay – Biological Replicate **2 (A)** Representative images obtained by confocal microscopy, using bright field (BF) and detection of GFP labelled cells at day 1 (D1) and after 8 days (D8) in culture for shWT (left) and shERK3 (right) spheroids. (Scale bar: 500 µm). **(B)** Representative confocal images of sectioned samples obtained from the same confrontation assay, cultured for 8 days. The cryosections were stained with DAPI (blue) and WGA (red). (Scale bar: 500 µm). **(C)** Representative images of the spheroids, highlighted by orange dashed line, the adhering area (dashed blue line) and penetrating cells (in dashed green line). (Scale bar: 250 µm). Spheroids are numbered across the biological replicates, in shWT and shERK3, from 1 to 11 or 1 to 12, respectively. Based on observation, they are colour coded in migrating spheroids (yellow), non-migrating spheroids (blue), or spheroids placed in direct contact with the LO (red). Same spheroids identified in panel A, are identified respectively in panel B and C.

Supplementary Figure 9.3 Confrontation Assay – Biological Replicate 3 **(A)** Representative images obtained by confocal microscopy, using bright field (BF) and detection of GFP labelled cells at day 1 (D1) and after 6 days (D6) in culture for shWT (left) and shERK3 (right) spheroids. (Scale bar: 500 µm). **(B)** Representative confocal images of sectioned samples obtained from the same confrontation assay, cultured for 6 days. The cryosections were stained with DAPI (blue) and WGA (red). It is also highlighted a region with a deep penetrating cell, which is identified by the letter “c” and arrowhead (Scale bar: 500 µm). **(C)** Representative images of the spheroids, highlighted by orange dashed line, the adhering area (dashed blue line) and penetrating cells (in dashed green line). (Scale bar: 250 µm). Spheroids are numbered across the biological replicates, in shWT and shERK3, from 1 to 11 or 1 to 12, respectively. Based on observation, they are colour coded in migrating spheroids (yellow), non-migrating spheroids (blue), or spheroids placed in direct contact with the LO (red). Same spheroids identified in panel **(A)**, are identified respectively in panel **(B, C)**.

Supplementary Figure 9.4 Confrontation Assay – Biological Replicate 4 **(A)** Representative images obtained by confocal microscopy, using bright field (BF) and detection of GFP labelled cells at day 1 (D1) and after 6 days (D6) in culture for shWT (left) and shERK3 (right) spheroids. (Scale bar: 500 µm). **(B)** Representative confocal images of sectioned samples obtained from the same confrontation assay, cultured for 6 days. The cryosections were stained with DAPI (blue) and WGA (red). It is also identified a region of a cluster of cells that adhered to the LO during culture with letter “c”. (Scale bar: 500 µm). **(C)** Representative images of the spheroids, highlighted by orange dashed line, the adhering area (dashed blue line) and penetrating cells (in dashed green line). (Scale bar: 250 µm). Spheroids are numbered across the biological replicates, in shWT and shERK3, from 1 to 11 or 1 to 12, respectively. Based on observation, they are colour coded in migrating spheroids (yellow), non-migrating spheroids (blue), or spheroids placed in direct contact with the LO (red). Same spheroids identified in panel **(A)**, are identified respectively in panel **(B, C)**.

Supplementary Datasheet 1 Western Blots - Uncropped Western blot membranes and corresponding Stain-Free blot acquired images.

## References

[1] Carey LA Dees EC Sawyer L Gatti L Moore DT Collichio F. The triple negative paradox: primary tumor chemosensitivity of breast cancer subtypes. Clin Cancer Res. (2007) 13:2329–34. doi: 10.1158/1078-0432.ccr-06-1109, PMID: 17438091

[2] Lüönd F Sugiyama N Bill R Bornes L Hager C Tang F. Distinct contributions of partial and full EMT to breast cancer Malignancy. Dev Cell. (2021) 56:3203–3221.e11. doi: 10.1016/j.devcel.2021.11.006, PMID: 34847378

[3] Yang J Antin P Berx G Blanpain C Brabletz T Bronner M. Guidelines and definitions for research on epithelial–mesenchymal transition. Nat Rev Mol Cell Biol. (2020) 21:341–52. doi: 10.1038/s41580-020-0237-9, PMID: 32300252 PMC7250738

[4] Pastushenko I Blanpain C Transition States during Tumor Progression EMT. and metastasis. Trends Cell Biol. (2019) 29:212–26. doi: 10.1016/j.tcb.2018.12.001, PMID: 30594349

[5] Cheung KJ Gabrielson E Werb Z Ewald AJ. Collective invasion in breast cancer requires a conserved basal epithelial program. Cell.(2013) 155:1639–51. doi: 10.1016/j.cell.2013.11.029, PMID: 24332913 PMC3941206

[6] Gui T Sun Y Shimokado A Muragaki Y. The roles of mitogen-activated protein kinase pathways in TGF- *β* -induced epithelial-mesenchymal transition. J Signal Transduction. (2012) 2012:1–10. doi: 10.1155/2012/289243, PMID: 22363839 PMC3272823

[7] Wang J Kuiatse I Lee AV Pan J Giuliano A Cui X. Sustained c-jun-NH2-kinase activity promotes epithelial-mesenchymal transition, invasion, and survival of breast cancer cells by regulating extracellular signal-regulated kinase activation. Mol Cancer Res. (2010) 8:266–77. doi: 10.1158/1541-7786.mcr-09-0221, PMID: 20145041 PMC2824784

[8] Bhatt AB Wright TD Barnes V Chakrabarty S Matossian MD Lexner E. Diverse and converging roles of ERK1/2 and ERK5 pathways on mesenchymal to epithelial transition in breast cancer. Transl Oncol. (2021) 14:101046. doi: 10.1016/j.tranon.2021.101046, PMID: 33761370 PMC8020482

[9] Antoon JW Nitzchke AM Martin EC Rhodes LV Nam S Wadsworth S. Inhibition of p38 mitogen-activated protein kinase alters microRNA expression and reverses epithelial-to-mesenchymal transition. Int J Oncol. (2013) 42:1139–50. doi: 10.3892/ijo.2013.1814, PMID: 23403951 PMC3622654

[10] Seternes OM Mikalsen T Johansen B Michaelsen E Armstrong CG Morrice NA. Activation of MK5/PRAK by the atypical MAP kinase ERK3 defines a novel signal transduction pathway. EMBO J. (2004) 23:4780–91. doi: 10.1038/sj.emboj.7600489, PMID: 15577943 PMC535098

[11] Bogucka K Marini F Rosigkeit S Schloeder J Jonuleit H David K. ERK3/MAPK6 is required for KRAS-mediated NSCLC tumorigenesis. Cancer Gene Ther. (2020). 28:359–74. doi: 10.1038/s41417-020-00245-w, PMID: 33070159

[12] Coulombe P Meloche S. Atypical mitogen-activated protein kinases: Structure, regulation and functions. Biochim Biophys Acta BBA - Mol Cell Res. (2007) 1773:1376–87. doi: 10.1016/j.bbamcr.2006.11.001, PMID: 17161475

[13] Vallabhaneni S Liu J Morel M Wang J DeMayo FJ Long W. Conditional *ERK3* overexpression cooperates with *PTEN* deletion to promote lung adenocarcinoma formation in mice. Mol Oncol. (2022) 16:1184–99. doi: 10.1002/1878-0261.13132, PMID: 34719109 PMC8895443

[14] Bogucka K Pompaiah M Marini F Binder H Harms G Kaulich M. ERK3/MAPK6 controls IL-8 production and chemotaxis. eLife.(2020) 9:e52511. doi: 10.7554/elife.52511, PMID: 32314963 PMC7192585

[15] Bogucka-Janczi K Harms G Coissieux MM Bentires-Alj M Thiede B Rajalingam K. ERK3/MAPK6 dictates CDC42/RAC1 activity and ARP2/3-dependent actin polymerization. eLife.(2023) 12:e85167. doi: 10.7554/elife.85167, PMID: 37057894 PMC10191626

[16] Cai Q Zhou W Wang W Dong B Han D Shen T. MAPK6-AKT signaling promotes tumor growth and resistance to mTOR kinase blockade. Sci Adv. (2021) 7:eabi6439. doi: 10.1126/sciadv.abi6439, PMID: 34767444 PMC8589317

[17] Liang B. Increased expression of mitogen-activated protein kinase and its upstream regulating signal in human gastric cancer. World J Gastroenterol. (2005) 11:623. doi: 10.3748/wjg.v11.i5.623, PMID: 15655810 PMC4250727

[18] Al-Mahdi R Babteen N Thillai K Holt M Johansen B Wetting HL. A novel role for atypical MAPK kinase ERK3 in regulating breast cancer cell morphology and migration. Cell Adhes Migr.(2015) 9:483–94. doi: 10.1080/19336918.2015.1112485, PMID: 26588708 PMC4955959

[19] Edgar R. Gene Expression Omnibus: NCBI gene expression and hybridization array data repository. Nucleic Acids Res. (2002) 30:207–10. doi: 10.1093/nar/30.1.207, PMID: 11752295 PMC99122

[20] Lonsdale J Thomas J Salvatore M Phillips R Lo E Shad S. The genotype-tissue expression (GTEx) project. Nat Genet. (2013) 45:580–5. doi: 10.1038/ng.2653, PMID: 23715323 PMC4010069

[21] The Cancer Genome Atlas Research Network JN W EA C GB M Shaw KRM BA O. The Cancer Genome Atlas Pan-Cancer analysis project. Nat Genet. (2013) 45:1113–20. doi: 10.1038/ng.2764, PMID: 24071849 PMC3919969

[22] Grossman RL Heath AP Ferretti V Varmus HE Lowy DR Kibbe WA. Toward a shared vision for cancer genomic data. N Engl J Med. (2016) 375:1109–12. doi: 10.1056/nejmp1607591, PMID: 27653561 PMC6309165

[23] Bartha Takoyaki San Daet llÁ Győrffy B. TNMplot.com: A web tool for the comparison of gene expression in normal, tumor and metastatic tissues. Int J Mol Sci. (2021) 22:2622. Available online at: https://tnmplot.com/ (Accessed June 13, 2022)., PMID: 33807717 10.3390/ijms22052622 PMC7961455

[24] Park SJ Yoon BH Kim SK Kim SY. GENT2: an updated gene expression database for normal and tumor tissues. BMC Med Genomics. (2019) 12:101. Available online at: http://gent2.appex.kr/gent2/ (Accessed June 27, 2022)., PMID: 31296229 10.1186/s12920-019-0514-7 PMC6624177

[25] Gyorffy Balázs. Transcriptome-level discovery of survival-associated biomarkers and therapy targets in non-small-cell lung cancer. Br J Pharmacol. (2023) 181:362–74. Available online at: https://kmplot.com/analysis (Accessed September 2, 2022)., PMID: 37783508 10.1111/bph.16257

[26] Jose SS De Zuani M Tidu F Hortová Kohoutková M Pazzagli L Forte G. Comparison of two human organoid models of lung and intestinal inflammation reveals Toll-like receptor signalling activation and monocyte recruitment. Clin Transl Immunol. (2020) 9:e1131. doi: 10.1002/cti2.1131, PMID: 32377340 PMC7200218

[27] Yu J Hu K Smuga-Otto K Tian S Stewart R Slukvin II. Human induced pluripotent stem cells free of vector and transgene sequences. Science.(2009) 324:797–801. doi: 10.1126/science.1172482, PMID: 19325077 PMC2758053

[28] Dye BR Hill DR Ferguson MA Tsai YH Nagy MS Dyal R. *In vitro* generation of human pluripotent stem cell derived lung organoids. eLife.(2015) 4:e05098. doi: 10.7554/elife.05098, PMID: 25803487 PMC4370217

[29] Miller AJ Dye BR Ferrer-Torres D Hill DR Overeem AW Shea LD. Generation of lung organoids from human pluripotent stem cells *in vitro* . Nat Protoc. (2019) 14:518–40. doi: 10.1038/s41596-018-0104-8, PMID: 30664680 PMC6531049

[30] Grädler U. Biochemical, cellular and structural characterization of novel and selective ERK3 inhibitors. Bioorg. Med Chem Lett. (2020) 30:127551. doi: 10.1016/j.bmcl.2020.127551, PMID: 32927028

[31] Javary J Goupil E Soulez M Kanshin E Bouchard A Seternes O. Phosphoproteomic analysis identifies supervillin as an ERK3 substrate regulating cytokinesis and cell ploidy. J Cell Physiol. (2024) 239:e30938. doi: 10.1002/jcp.30938, PMID: 36576983

[32] Campeau E Ruhl VE Rodier F Smith CL Rahmberg BL Fuss JO. A versatile viral system for expression and depletion of proteins in mammalian cells. PloS One. (2009) 4:e6529. doi: 10.1371/journal.pone.0006529, PMID: 19657394 PMC2717805

[33] Suarez-Arnedo A Torres Figueroa F Clavijo C Arbeláez P Cruz JC Muñoz-Camargo C. An image J plugin for the high throughput image analysis of *in vitro* scratch wound healing assays. PloS One. (2020) 15:e0232565. doi: 10.1371/journal.pone.0232565, PMID: 32722676 PMC7386569

[34] Haase K Gillrie MR Hajal C Kamm RD. Pericytes contribute to dysfunction in a human 3D model of placental microvasculature through VEGF-ang-tie2 signaling. Adv Sci. (2019) 6:1900878. doi: 10.1002/advs.201900878, PMID: 31832308 PMC6891921

[35] Chen MB Whisler JA Fröse J Yu C Shin Y Kamm RD. On-chip human microvasculature assay for visualization and quantification of tumor cell extravasation dynamics. Nat Protoc. (2017) 12:865–80. doi: 10.1038/nprot.2017.018, PMID: 28358393 PMC5509465

[36] Wu Y Li C Peng H Swaidan A Riehle A Pollmeier B. Acid sphingomyelinase contributes to the control of mycobacterial infection via a signaling cascade leading from reactive oxygen species to cathepsin D. Cells.(2020) 9:2406. doi: 10.3390/cells9112406, PMID: 33153072 PMC7693114

[37] Srivastava GK Reinoso R Singh AK Fernandez-Bueno I Hileeto D Martino M. Trypan Blue staining method for quenching the autofluorescence of RPE cells for improving protein expression analysis. Exp Eye Res. (2011) 93:956–62. doi: 10.1016/j.exer.2011.07.002, PMID: 21777584

[38] Cowen T Haven AJ Burnstock G. Pontamine sky blue: A counterstain for background autofluorescence in fluorescence and immunofluorescence histochemistry. Histochemistry.(1985) 82:205–8. doi: 10.1007/bf00501396, PMID: 2581921

[39] Nolan E Lindeman GJ Visvader JE. Deciphering breast cancer: from biology to the clinic. Cell.(2023) 186:1708–28. doi: 10.1016/j.cell.2023.01.040, PMID: 36931265

[40] Lehmann BD Bauer JA Chen X Sanders ME Chakravarthy AB Shyr Y. Identification of human triple-negative breast cancer subtypes and preclinical models for selection of targeted therapies. J Clin Invest.(2011) 121:2750–67. doi: 10.1172/jci45014, PMID: 21633166 PMC3127435

[41] Lakhani SR Ellis IO Schnitt SJ Tan PH. WHO classification of tumours of the breast. International Agency for Research on Cancer (IARC) 69372 Lyon Cedex 08, France. (2012).

[42] Surveillance, epidemiology, and end results program. In: National institute of health (NIH) - cancer stat facts: female breast cancer subtypes. Available online at: https://seer.cancer.gov/statfacts/html/breast-subtypes.html (Accessed February 21, 2023).

[43] Surveillance, epidemiology, and end results program (SEER) . Available online at: https://seer.cancer.gov (Accessed Feb 7, 2024).

[44] Neve RM Chin K Fridlyand J Yeh J Baehner FL Fevr T. A collection of breast cancer cell lines for the study of functionally distinct cancer subtypes. Cancer Cell. (2006) 10:515–27. doi: 10.1016/j.ccr.2006.10.008, PMID: 17157791 PMC2730521

[45] Vilchez Mercedes SA Bocci F Levine H Onuchic JN Jolly MK Wong PK. Decoding leader cells in collective cancer invasion. Nat Rev Cancer.(2021) 21:592–604. doi: 10.1038/s41568-021-00376-8, PMID: 34239104

[46] Insua-Rodríguez J Oskarsson T. The extracellular matrix in breast cancer. Adv Drug Delivery Rev. (2016) 97:41–55. doi: 10.1016/j.addr.2015.12.017, PMID: 26743193

[47] Elkhadragy L Myers A Long W. Role of the atypical MAPK ERK3 in cancer growth and progression. Cancers.(2024) 16:1381. doi: 10.3390/cancers16071381, PMID: 38611058 PMC11011113

[48] Elkhadragy L Alsaran H Long W. The C-terminus tail regulates ERK3 kinase activity and its ability in promoting cancer cell migration and invasion. Int J Mol Sci. (2020) 21:4044. doi: 10.3390/ijms21114044, PMID: 32516969 PMC7312006

[49] Elkhadragy L Alsaran H Morel M Long W. Activation loop phosphorylation of ERK3 is important for its kinase activity and ability to promote lung cancer cell invasiveness. J Biol Chem. (2018) 293:16193–205. doi: 10.1074/jbc.ra118.003699, PMID: 30166347 PMC6200930

[50] Strilic B Offermanns S. Intravascular survival and extravasation of tumor cells. Cancer Cell. (2017) 32:282–93. doi: 10.1016/j.ccell.2017.07.001, PMID: 28898694

[51] Burstein HJ Curigliano G Thürlimann B Weber WP Poortmans P Regan MM. Customizing local and systemic therapies for women with early breast cancer: the St. Gallen International Consensus Guidelines for treatment of early breast cancer 2021. Ann Oncol. (2021) 32:1216–35. doi: 10.1016/j.annonc.2021.06.023, PMID: 34242744 PMC9906308

[52] Kröger C Afeyan A Mraz J Eaton EN Reinhardt F Khodor YL. Acquisition of a hybrid E/M state is essential for tumorigenicity of basal breast cancer cells. Proc Natl Acad Sci. (2019) 116:7353–62. doi: 10.1073/pnas.1812876116, PMID: 30910979 PMC6462070

[53] Pastushenko I Brisebarre A Sifrim A Fioramonti M Revenco T Boumahdi S. Identification of the tumour transition states occurring during EMT. Nature.(2018) 556:463–8. doi: 10.1038/s41586-018-0040-3, PMID: 29670281

[54] Tang T Zhu Q Li X Zhu G Deng S Wang Y. Protease Nexin I is a feedback regulator of EGF/PKC/MAPK/EGR1 signaling in breast cancer cells metastasis and stemness. Cell Death Dis. (2019) 10:649. doi: 10.1038/s41419-019-1882-9, PMID: 31501409 PMC6733841

[55] Zhu C Kong Z Wang B Cheng W Wu A Meng X. ITGB3/CD61: a hub modulator and target in the tumor microenvironment. Am J Transl Res. (2019) 11(12):7195–208., PMID: 31934272 PMC6943458

[56] Tulotta C Lefley DV Moore CK Amariutei AE Spicer-Hadlington AR Quayle LA. IL-1B drives opposing responses in primary tumours and bone metastases; harnessing combination therapies to improve outcome in breast cancer. NPJ Breast Cancer.(2021) 7:95. doi: 10.1038/s41523-021-00305-w, PMID: 34290237 PMC8295314

[57] Chang CM Lam HP Hsu HJ Jiang SJ. Interleukin-10: A double-edged sword in breast cancer. Tzu Chi Med J. (2021) 33:203. doi: 10.4103/tcmj.tcmj_162_20, PMID: 34386356 PMC8323643

[58] Moore KM Thomas GJ Duffy SW Warwick J Gabe R Chou P. Therapeutic targeting of integrin αvβ6 in breast cancer. JNCI J Natl Cancer Inst. (2014) 106. doi: 10.1093/jnci/dju169, PMID: 24974129 PMC4151855

[59] Cheng G Fan X Hao M Wang J Zhou X Sun X. Higher levels of TIMP-1 expression are associated with a poor prognosis in triple-negative breast cancer. Mol Cancer.(2016) 15:30. doi: 10.1186/s12943-016-0515-5, PMID: 27130446 PMC4851824

[60] Banys-Paluchowski M Witzel I Aktas B Fasching PA Hartkopf A Janni W. The prognostic relevance of urokinase-type plasminogen activator (uPA) in the blood of patients with metastatic breast cancer. Sci Rep. (2019) 9:2318. doi: 10.1038/s41598-018-37259-2, PMID: 30783124 PMC6381129

[61] Wang MY Chen PS Prakash E Hsu HC Huang HY Lin MT. Connective Tissue Growth Factor Confers Drug Resistance in Breast Cancer through Concomitant Up-regulation of Bcl-xL and cIAP1. Cancer Res. (2009) 69:3482–91. doi: 10.1158/0008-5472.can-08-2524, PMID: 19351859

[62] Su X Xu Y Fox GC Xiang J Kwakwa KA Davis JL. Breast cancer–derived GM-CSF regulates arginase 1 in myeloid cells to promote an immunosuppressive microenvironment. J Clin Invest.(2021) 131:e145296. doi: 10.1172/jci145296, PMID: 34520398 PMC8516467

[63] Liu L Wu Y Zhang C Zhou C Li Y Zeng Y. Cancer-associated adipocyte-derived G-CSF promotes breast cancer Malignancy via Stat3 signaling. J Mol Cell Biol. (2020) 12:723–37. doi: 10.1093/jmcb/mjaa016, PMID: 32242230 PMC7749739

[64] Liu Y Song Y Ye M Hu X Wang ZP Zhu X. The emerging role of WISP proteins in tumorigenesis and cancer therapy. J Transl Med. (2019) 17:28. doi: 10.1186/s12967-019-1769-7, PMID: 30651114 PMC6335850

[65] Wang S Li J Hong S Wang N Xu S Yang B. Chemotherapy-elicited extracellular vesicle CXCL1 from dying cells promotes triple-negative breast cancer metastasis by activating TAM/PD-L1 signaling. J Exp Clin Cancer Res. (2024) 43:121. doi: 10.1186/s13046-024-03050-7, PMID: 38654356 PMC11036662

[66] Sereesongsaeng N McDowell SH Burrows JF Scott CJ Burden RE. Cathepsin V suppresses GATA3 protein expression in luminal A breast cancer. Breast Cancer Res. (2020) 22:139. doi: 10.1186/s13058-020-01376-6, PMID: 33298139 PMC7726886

[67] SenGupta S Hein LE Xu Y Zhang J Konwerski JR Li Y. Triple-negative breast cancer cells recruit neutrophils by secreting TGF-β and CXCR2 ligands. Front Immunol. (2021) 12:659996. doi: 10.3389/fimmu.2021.659996, PMID: 33912188 PMC8071875

[68] Ibrahim SA El-Ghonaimy EA Hassan H Mahana N Mahmoud MA El-Mamlouk T. Hormonal-receptor positive breast cancer: IL-6 augments invasion and lymph node metastasis via stimulating cathepsin B expression. J Adv Res. (2016) 7:661–70. doi: 10.1016/j.jare.2016.06.007, PMID: 27482469 PMC4957008

[69] Kim SH Ryu KJ Hong KS Kim H Han H Kim M. ERK3 increases snail protein stability by inhibiting FBXO11-mediated snail ubiquitination. Cancers.(2023) 16:105. doi: 10.3390/cancers16010105, PMID: 38201533 PMC10777929

[70] Boussommier-Calleja A Atiyas Y Haase K Headley M Lewis C Kamm RD. The effects of monocytes on tumor cell extravasation in a 3D vascularized microfluidic model. Biomaterials.(2019) 198:180–93. doi: 10.1016/j.biomaterials.2018.03.005, PMID: 29548546 PMC6123301

[71] Fernandes S Oliver-De La Cruz J Morazzo S Niro F Cassani M Ďuríková H. TGF-β induces matrisome pathological alterations and EMT in patient-derived prostate cancer tumoroids. Matrix Biol. (2024) 125:12–30. doi: 10.1016/j.matbio.2023.11.001, PMID: 37944712

[72] Mathien S Déléris P Soulez M Voisin L Meloche S. Deubiquitinating enzyme USP20 regulates extracellular signal-regulated kinase 3 stability and biological activity. Mol Cell Biol. (2017) 37:e00432–16. doi: 10.1128/mcb.00432-16, PMID: 28167606 PMC5394282

[73] Takahashi C Miyatake K Kusakabe M Nishida E. The atypical mitogen-activated protein kinase ERK3 is essential for establishment of epithelial architecture. J Biol Chem. (2018) 293:8342–61. doi: 10.1074/jbc.ra117.000992, PMID: 29674317 PMC5986203

[74] Huang Y Liu R Han X Hou X Tian Y Zhang W. Rab31 promotes the invasion and metastasis of cervical cancer cells by inhibiting MAPK6 degradation. Int J Biol Sci. (2022) 18:112–23. doi: 10.7150/ijbs.63388, PMID: 34975321 PMC8692139

[75] Expression of MAPK6 in cancer - Summary - The Human Protein Atlas . Available online at: https://www.proteinatlas.org/ENSG00000069956-MAPK6/pathology (Accessed Apr 14, 2021).

[76] Myers AK Morel M Gee SH Hoffmann KA Long W. ERK3 and DGKζ interact to modulate cell motility in lung cancer cells. Front Cell Dev Biol. (2023) 11:1192221. doi: 10.3389/fcell.2023.1192221, PMID: 37287450 PMC10242005

[77] Brand F Schumacher S Kant S Menon MB Simon R Turgeon B. The extracellular signal-regulated kinase 3 (Mitogen-activated protein kinase 6 [MAPK6])–MAPK-activated protein kinase 5 signaling complex regulates septin function and dendrite morphology. Mol Cell Biol. (2012) 32:2467–78. doi: 10.1128/mcb.06633-11, PMID: 22508986 PMC3434500

[78] Long W Foulds CE Qin J Liu J Ding C Lonard DM. ERK3 signals through SRC-3 coactivator to promote human lung cancer cell invasion. J Clin Invest.(2012) 122:1869–80. doi: 10.1172/jci61492, PMID: 22505454 PMC3336992

[79] Quinn HM Vogel R Popp O Mertins P Lan L Messerschmidt C. YAP and β-catenin cooperate to drive oncogenesis in basal breast cancer. Cancer Res. (2021) 81:2116–27. doi: 10.1158/0008-5472.can-20-2801, PMID: 33574090

[80] Luo J Zou H Guo Y Tong T Chen Y Xiao Y. The oncogenic roles and clinical implications of YAP/TAZ in breast cancer. Br J Cancer.(2023) 128:1611–24. doi: 10.1038/s41416-023-02182-5, PMID: 36759723 PMC10133323

[81] Xu L Corcoran RB Welsh JW Pennica D Levine AJ. WISP-1 is a Wnt-1- and -catenin- responsive oncogene. Genes Dev. (2000) 14(5):585–95., PMID: 10716946 PMC316421

[82] Zhong Q Qiu R. Mechanism of lncRNA SNHG19 miR-299-5p MAPK6 signaling axis promoting metastasis of non-small cell lung cancer cells. Oncol Transl Med. (2022) 8:247–58. doi: 10.1007/s10330-022-0595-5

[83] Huang YT Lan Q Lorusso G Duffey N Rüegg C. The matricellular protein CYR61 promotes breast cancer lung metastasis by facilitating tumor cell extravasation and suppressing anoikis. Oncotarget.(2017) 8:9200–15. doi: 10.18632/oncotarget.13677, PMID: 27911269 PMC5354725

[84] Espinoza I Kurapaty C Park CH Steen TV Kleer CG Wiley E. Depletion of CCN1/CYR61 reduces triple-negative/basal-like breast cancer aggressiveness. Am J Cancer Res. (2022) 12(2):839–51., PMID: 35261806 PMC8899977

