# Supplementary material for: ERK3/MAPK6 promotes triple-negative breast cancer progression through collective migration and EMT plasticity
Source: Front Oncol. 2025 Aug 27;15:1563969. doi: 10.3389/fonc.2025.1563969 (PMC12420279; doi:10.3389/fonc.2025.1563969)
Supplement: Supplementary Table 1 — Differentially regulated genes by ERK3, detected by RT² Profiler™ PCR Array Human Wound Healing (cut off values FC >1.5, P-value <0.05). The genes that passed the cut off threshold are highlighted in bold. [file Table1.docx]

**Table S1.**Differentially regulated genes by ERK3, detected by RT² Profiler™ PCR Array Human Wound Healing (cut off values FC >1.5, P-value <0.05). The genes that passed the cut off threshold are highlighted in bold.

| **Up-regulated in shWT** | | | **Up-regulated in shERK3** | | |
| --- | --- | --- | --- | --- | --- |
| Gene Symbol | Fold Regulation | P-value | Gene Symbol | Fold Regulation | P-value |
| **F13A1** | 6.63 | 0.00071 | **CSF3** | 8.02 | 0.000145 |
| **IL10** | 3.06 | 0.010336 | **IL1B** | 5.58 | 0.000258 |
| **CCN4** | 2.68 | 0.010144 | **MMP1** | 4.35 | 0.001449 |
| **CTSV** | 2.18 | 0.017913 | **PTGS2** | 3.95 | 0.000042 |
| **ITGB3** | 1.94 | 0.011791 | **FGF7** | 3.05 | 0.000885 |
| **CCN2** | 1.91 | 0.001323 | **CXCL11** | 2.56 | 0.00005 |
| **ITGB6** | 1.77 | 0.036327 | **TNF** | 2.56 | 0.003514 |
| **PTEN** | 1.65 | 0.002074 | **CDH1** | 2.51 | 0.001262 |
| **TIMP1** | 1.65 | 0.0001 | **MMP7** | 2.49 | 0.032942 |
| **PLAU** | 1.52 | 0.011425 | **CXCL1** | 2.3 | 0.000937 |
| **EGF** | 1.51 | 0.000526 | **IL6** | 2.02 | 0.003782 |
| MMP9 | 2.25 | 0.086438 | **CSF2** | 1.66 | 0.000194 |
| CCL2 | 2.11 | 0.074639 | **CXCL2** | 1.65 | 0.003222 |
| COL5A1 | 2 | 0.059602 | **PLAT** | 1.57 | 0.001047 |
