# Supplementary figures and images for: ERK3/MAPK6 promotes triple-negative breast cancer progression through collective migration and EMT plasticity
Source: Front Oncol. 2025 Aug 27;15:1563969. doi: 10.3389/fonc.2025.1563969 (PMC12420279; doi:10.3389/fonc.2025.1563969)

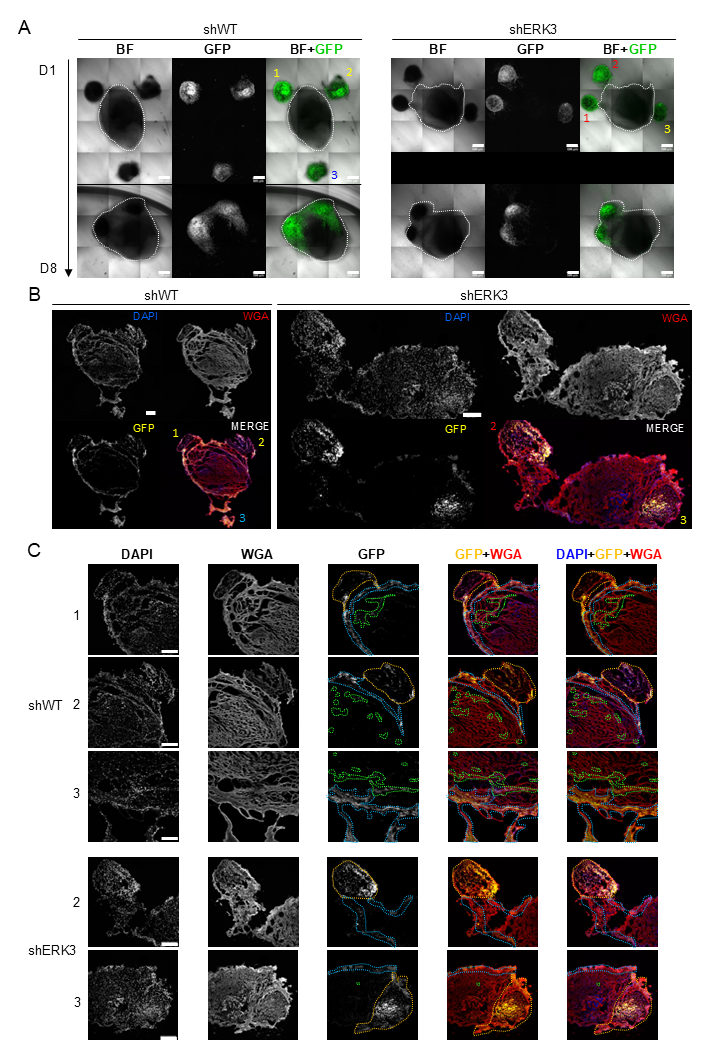

Supplement: Supplementary Figure 1 — ERK3 is overexpressed in primary tumour patient samples compared to normal tissue, adjacent to the tumour. Data, in transcripts per million (TPM), was extracted from the TNMplot database (tnmplot.com) and is shown as mean ± SD. Statistical analysis was performed with Wilcoxon matched pairs signed rank test. (N=70). ****P<0.0001 [file Presentation1.zip › Image 9.1.TIF]

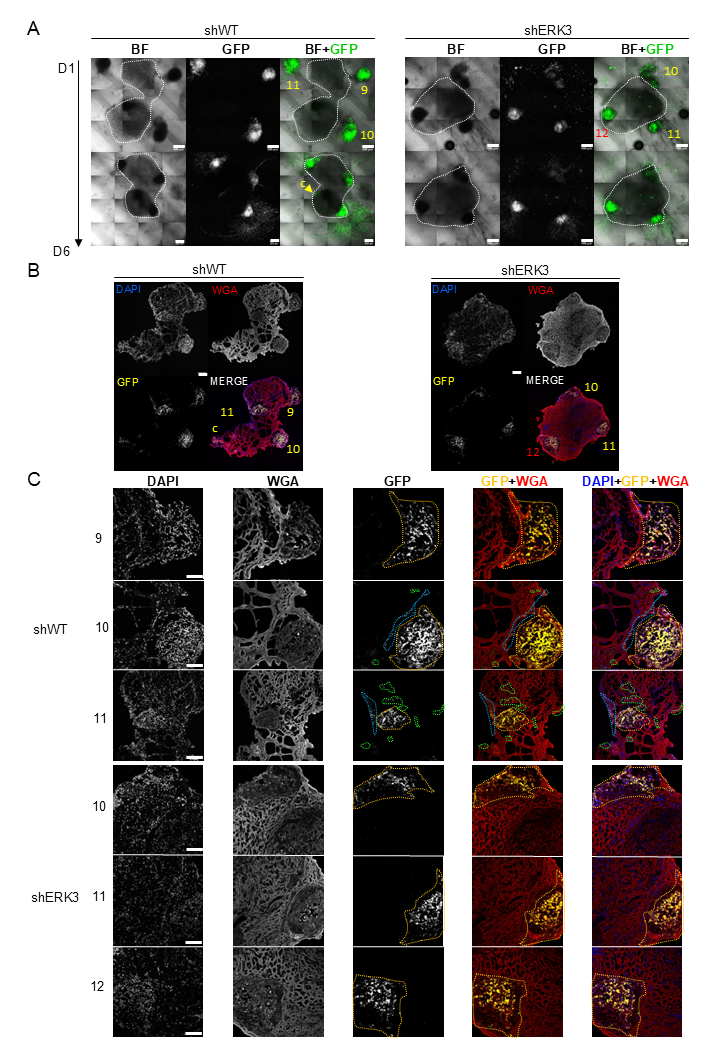

Supplement: Supplementary Figure 1 — ERK3 is overexpressed in primary tumour patient samples compared to normal tissue, adjacent to the tumour. Data, in transcripts per million (TPM), was extracted from the TNMplot database (tnmplot.com) and is shown as mean ± SD. Statistical analysis was performed with Wilcoxon matched pairs signed rank test. (N=70). ****P<0.0001 [file Presentation1.zip › Image 9.4.TIF]

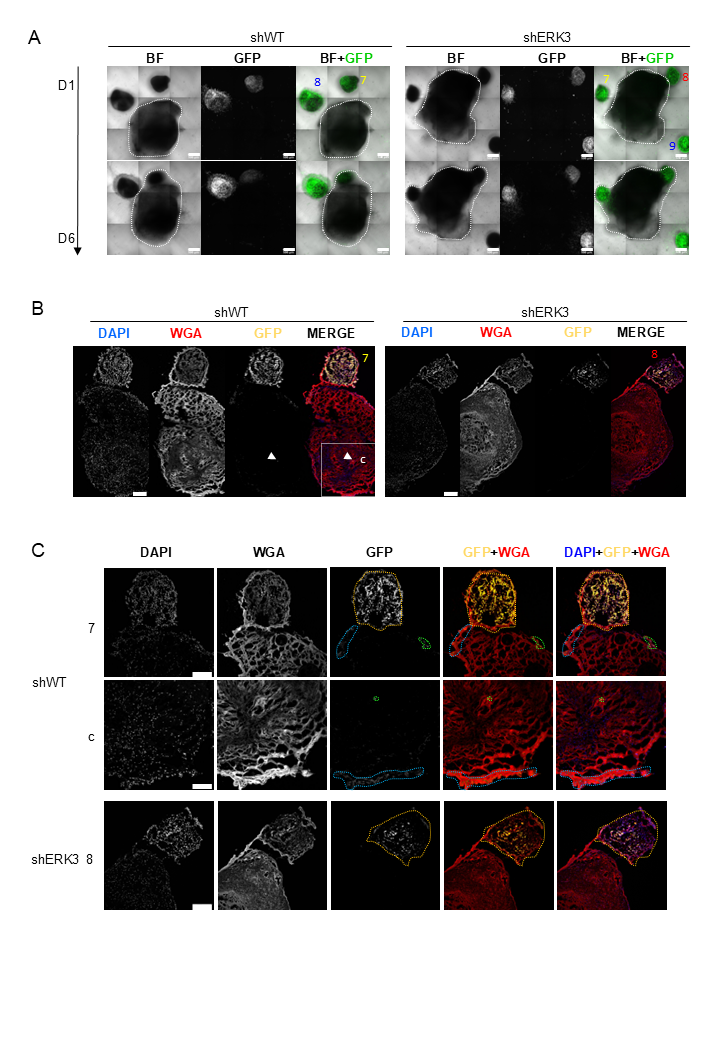

Supplement: Supplementary Figure 1 — ERK3 is overexpressed in primary tumour patient samples compared to normal tissue, adjacent to the tumour. Data, in transcripts per million (TPM), was extracted from the TNMplot database (tnmplot.com) and is shown as mean ± SD. Statistical analysis was performed with Wilcoxon matched pairs signed rank test. (N=70). ****P<0.0001 [file Presentation1.zip › Image 9.3.TIF]

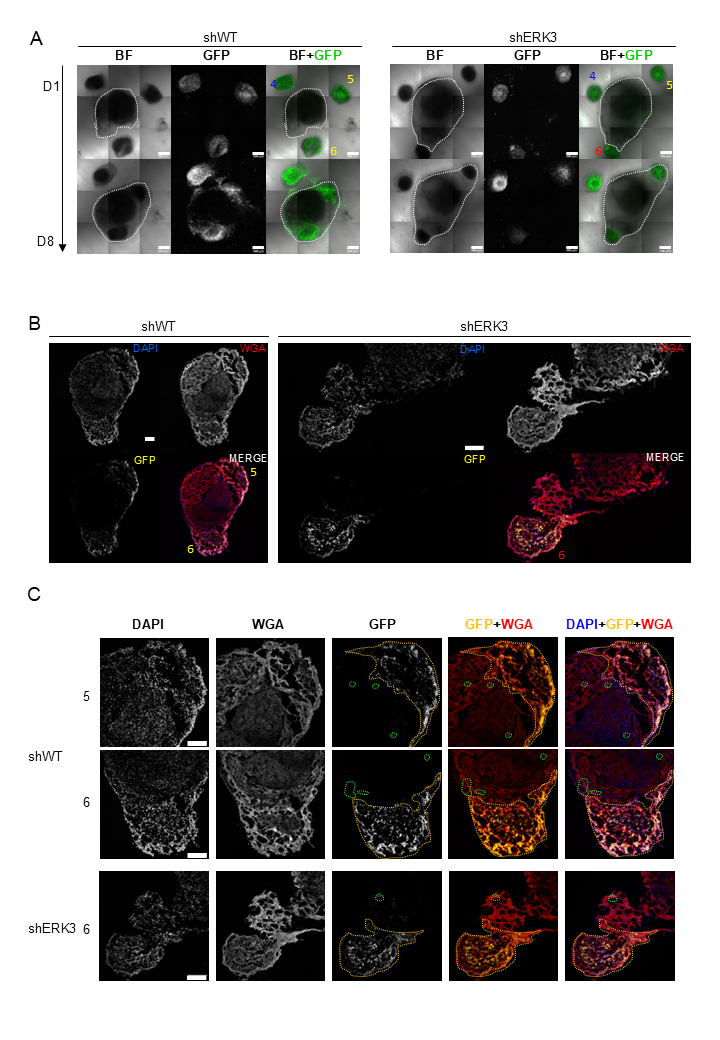

Supplement: Supplementary Figure 1 — ERK3 is overexpressed in primary tumour patient samples compared to normal tissue, adjacent to the tumour. Data, in transcripts per million (TPM), was extracted from the TNMplot database (tnmplot.com) and is shown as mean ± SD. Statistical analysis was performed with Wilcoxon matched pairs signed rank test. (N=70). ****P<0.0001 [file Presentation1.zip › Image 9.2.TIF]

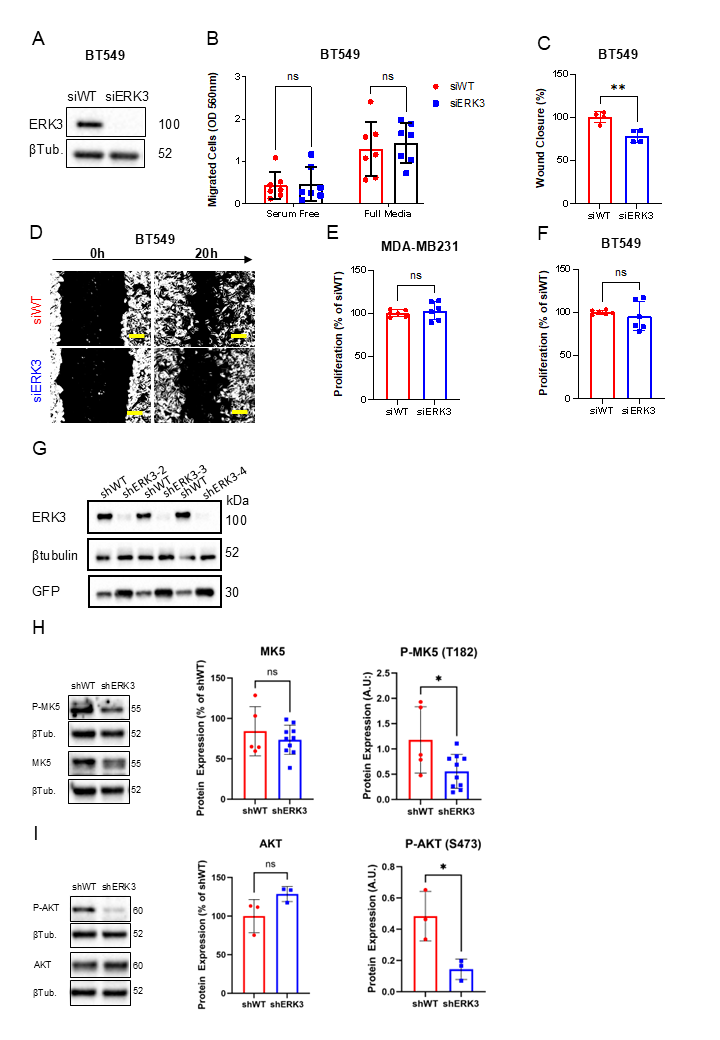

Supplement: Supplementary Figure 1 — ERK3 is overexpressed in primary tumour patient samples compared to normal tissue, adjacent to the tumour. Data, in transcripts per million (TPM), was extracted from the TNMplot database (tnmplot.com) and is shown as mean ± SD. Statistical analysis was performed with Wilcoxon matched pairs signed rank test. (N=70). ****P<0.0001 [file Presentation1.zip › Image 2.TIF]

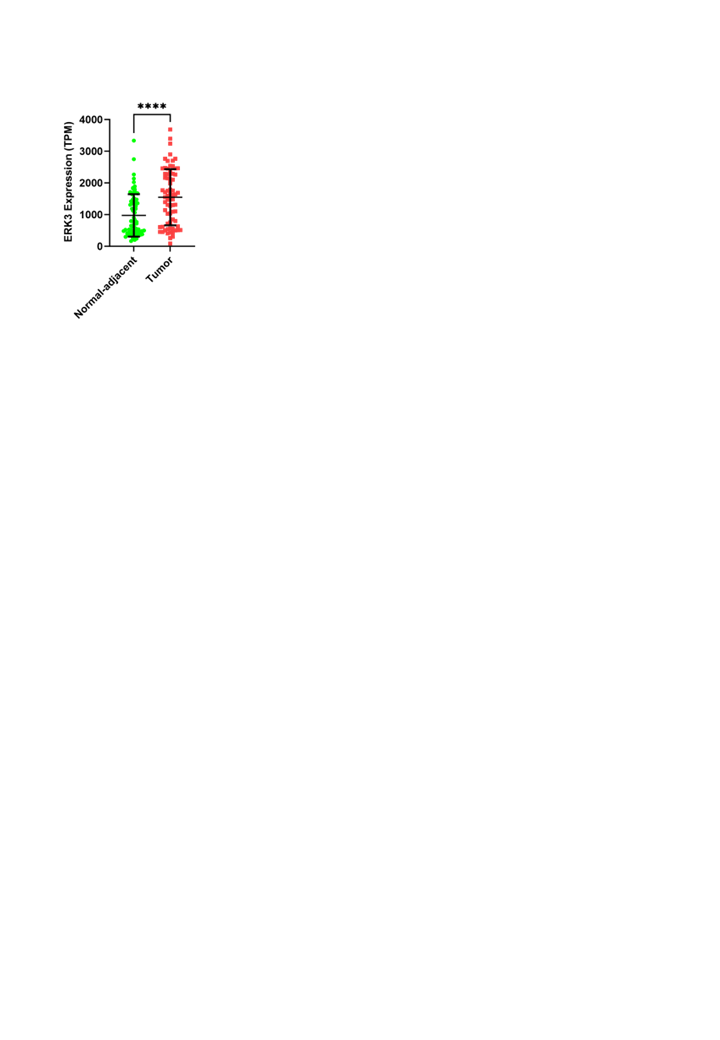

Supplement: Supplementary Figure 1 — ERK3 is overexpressed in primary tumour patient samples compared to normal tissue, adjacent to the tumour. Data, in transcripts per million (TPM), was extracted from the TNMplot database (tnmplot.com) and is shown as mean ± SD. Statistical analysis was performed with Wilcoxon matched pairs signed rank test. (N=70). ****P<0.0001 [file Presentation1.zip › Image 1.TIF]

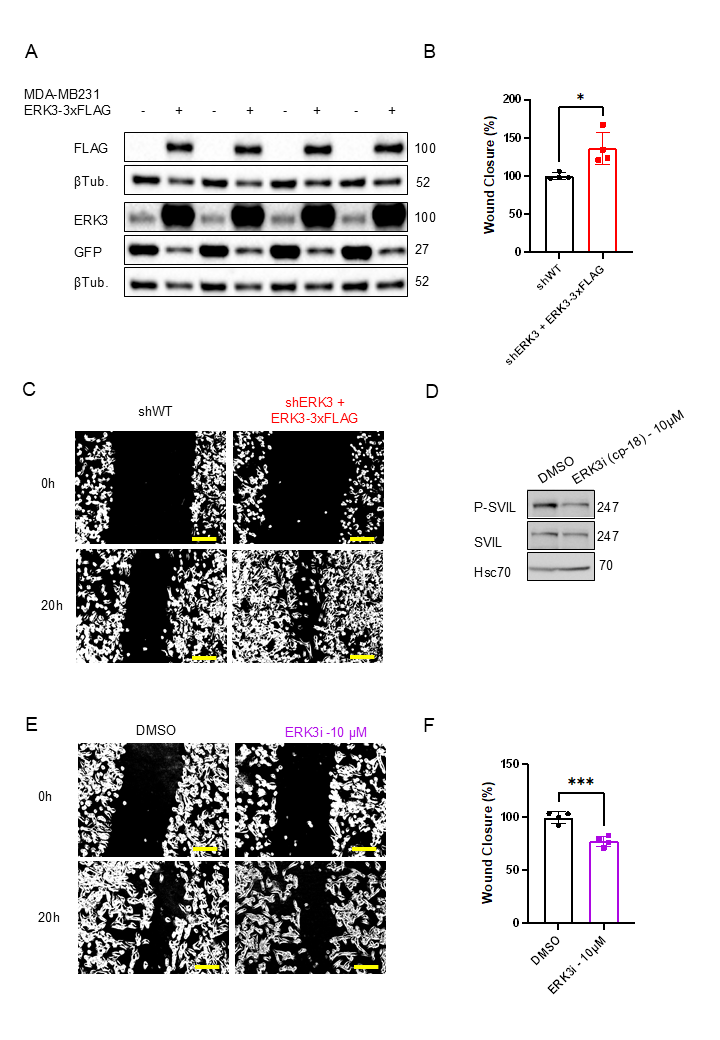

Supplement: Supplementary Figure 1 — ERK3 is overexpressed in primary tumour patient samples compared to normal tissue, adjacent to the tumour. Data, in transcripts per million (TPM), was extracted from the TNMplot database (tnmplot.com) and is shown as mean ± SD. Statistical analysis was performed with Wilcoxon matched pairs signed rank test. (N=70). ****P<0.0001 [file Presentation1.zip › Image 3.TIF]

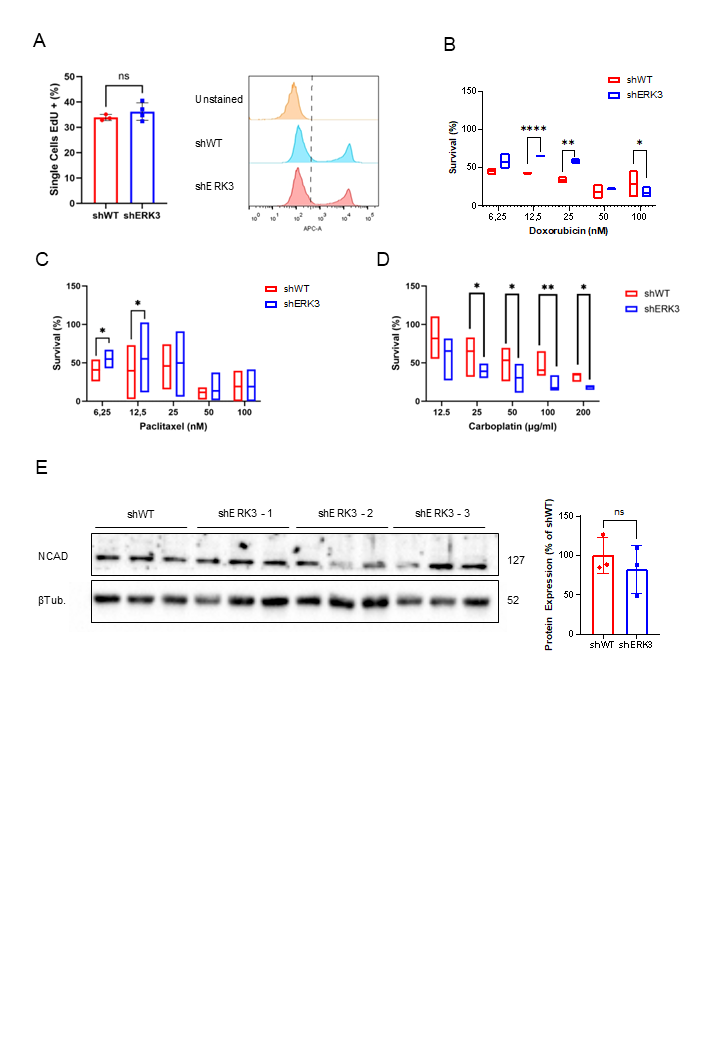

Supplement: Supplementary Figure 1 — ERK3 is overexpressed in primary tumour patient samples compared to normal tissue, adjacent to the tumour. Data, in transcripts per million (TPM), was extracted from the TNMplot database (tnmplot.com) and is shown as mean ± SD. Statistical analysis was performed with Wilcoxon matched pairs signed rank test. (N=70). ****P<0.0001 [file Presentation1.zip › Image 4.TIF]

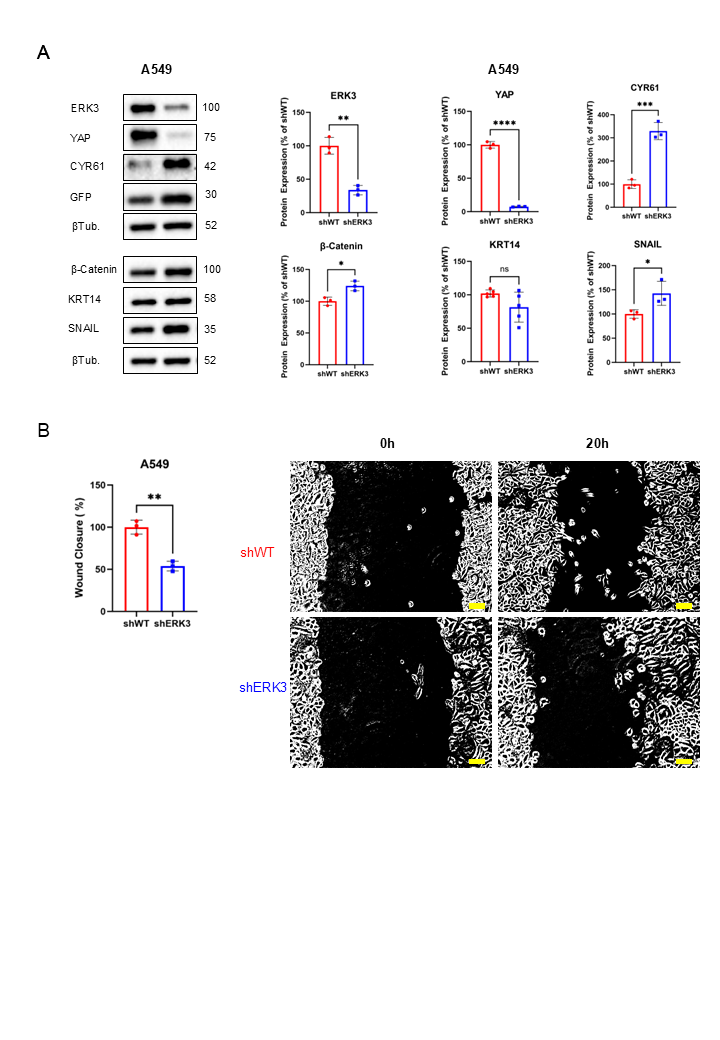

Supplement: Supplementary Figure 1 — ERK3 is overexpressed in primary tumour patient samples compared to normal tissue, adjacent to the tumour. Data, in transcripts per million (TPM), was extracted from the TNMplot database (tnmplot.com) and is shown as mean ± SD. Statistical analysis was performed with Wilcoxon matched pairs signed rank test. (N=70). ****P<0.0001 [file Presentation1.zip › Image 5.TIF]

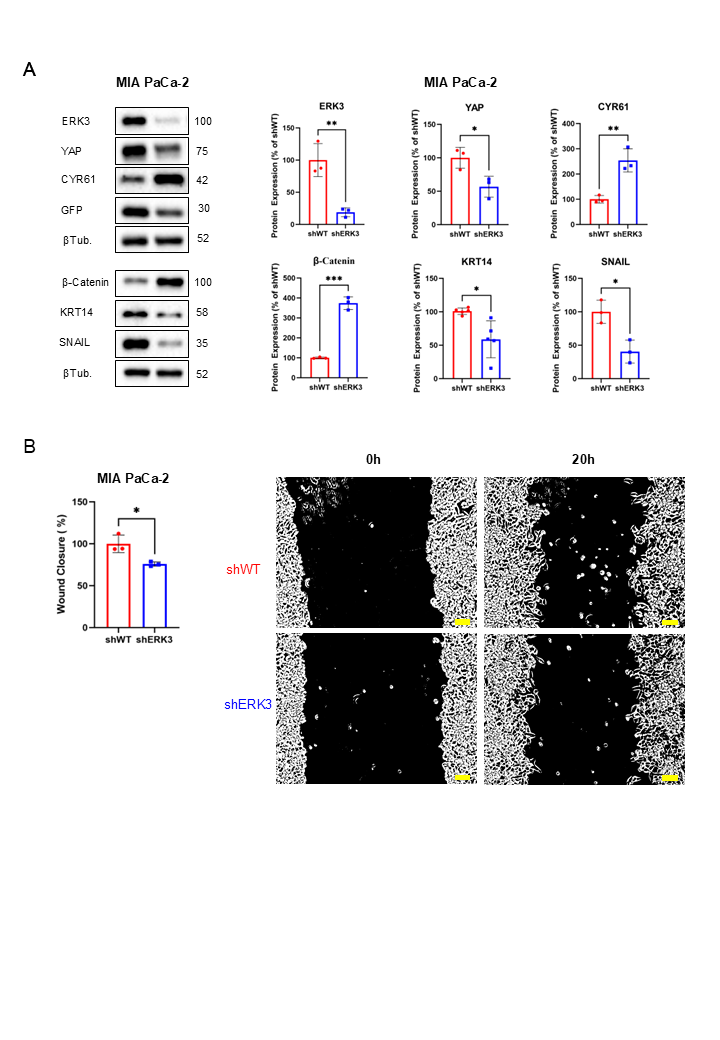

Supplement: Supplementary Figure 1 — ERK3 is overexpressed in primary tumour patient samples compared to normal tissue, adjacent to the tumour. Data, in transcripts per million (TPM), was extracted from the TNMplot database (tnmplot.com) and is shown as mean ± SD. Statistical analysis was performed with Wilcoxon matched pairs signed rank test. (N=70). ****P<0.0001 [file Presentation1.zip › Image 6.TIF]

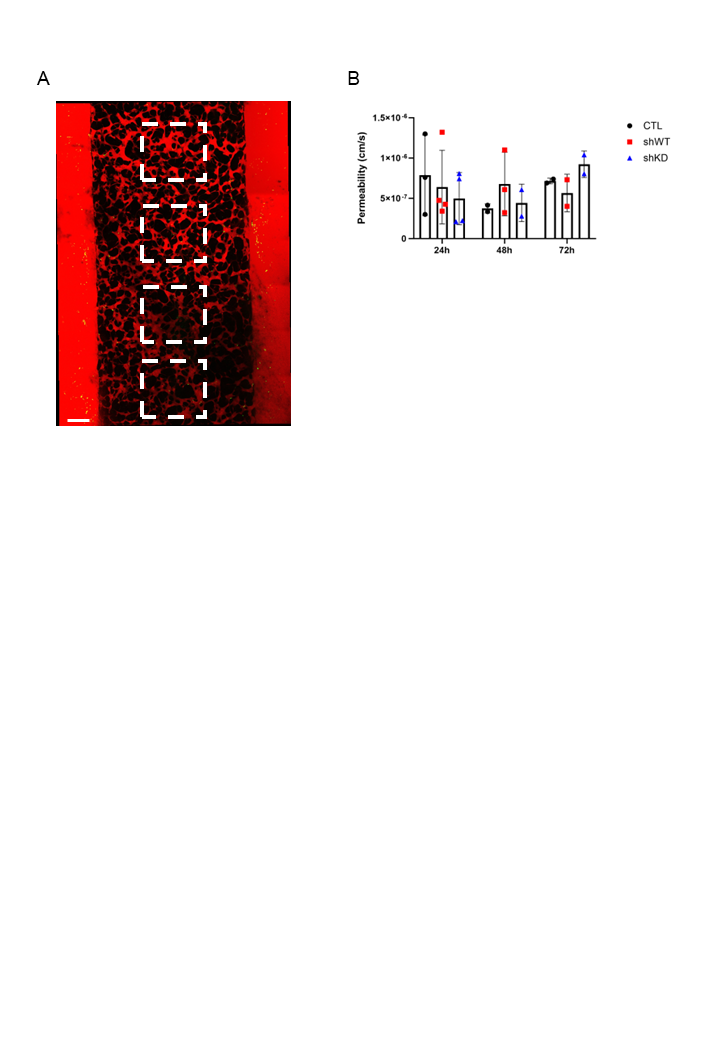

Supplement: Supplementary Figure 1 — ERK3 is overexpressed in primary tumour patient samples compared to normal tissue, adjacent to the tumour. Data, in transcripts per million (TPM), was extracted from the TNMplot database (tnmplot.com) and is shown as mean ± SD. Statistical analysis was performed with Wilcoxon matched pairs signed rank test. (N=70). ****P<0.0001 [file Presentation1.zip › Image 7.TIF]

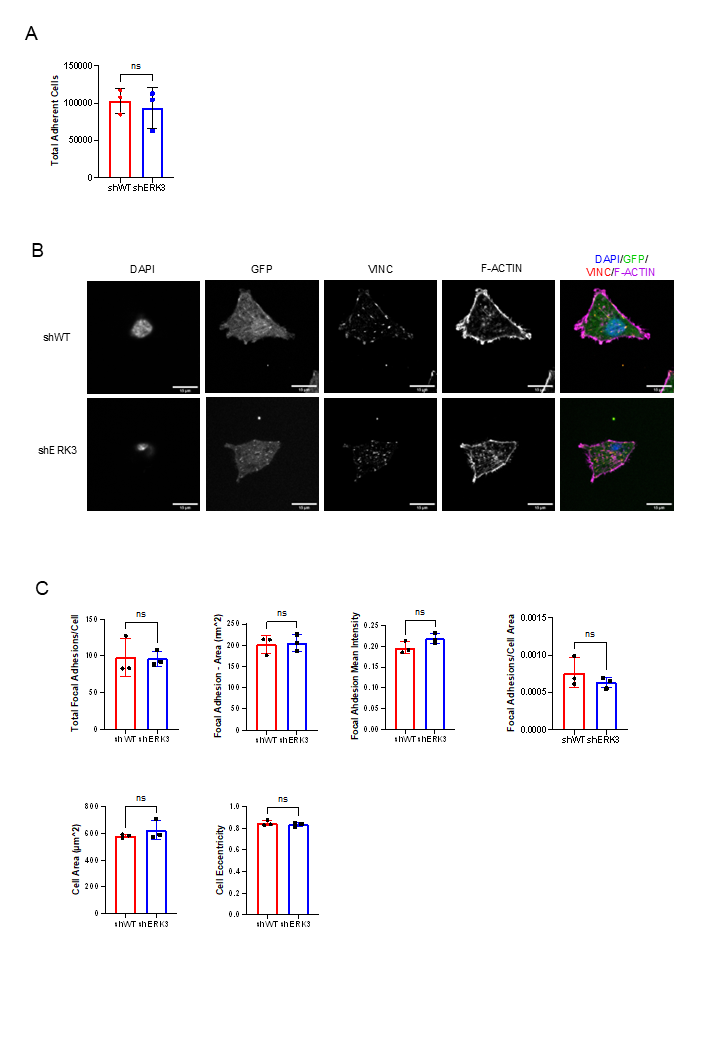

Supplement: Supplementary Figure 1 — ERK3 is overexpressed in primary tumour patient samples compared to normal tissue, adjacent to the tumour. Data, in transcripts per million (TPM), was extracted from the TNMplot database (tnmplot.com) and is shown as mean ± SD. Statistical analysis was performed with Wilcoxon matched pairs signed rank test. (N=70). ****P<0.0001 [file Presentation1.zip › Image 8.TIF]
