# Supplementary material for: ERK3/MAPK6 promotes triple-negative breast cancer progression through collective migration and EMT plasticity
Source: Front Oncol. 2025 Aug 27;15:1563969. doi: 10.3389/fonc.2025.1563969 (PMC12420279; doi:10.3389/fonc.2025.1563969)

Western Blot – Figure 2A

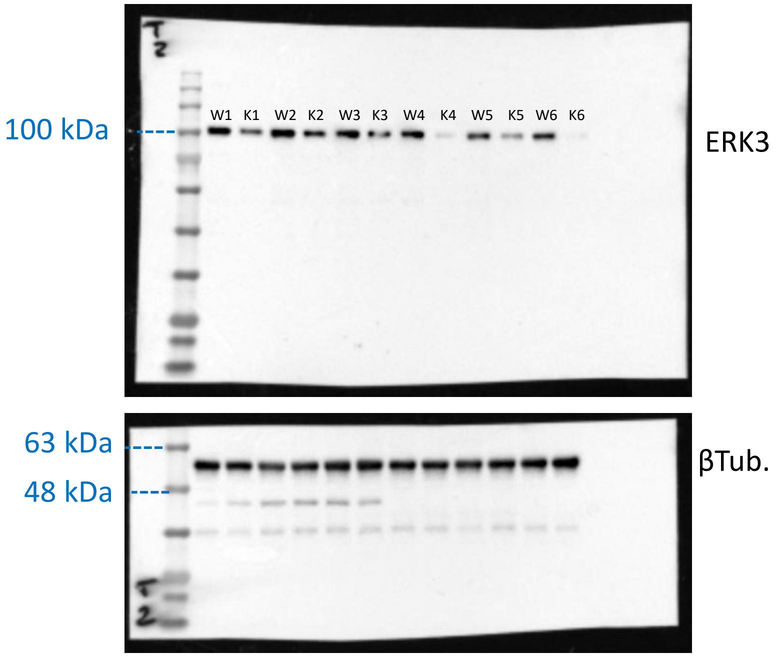

Corresponding Stain Free Blot

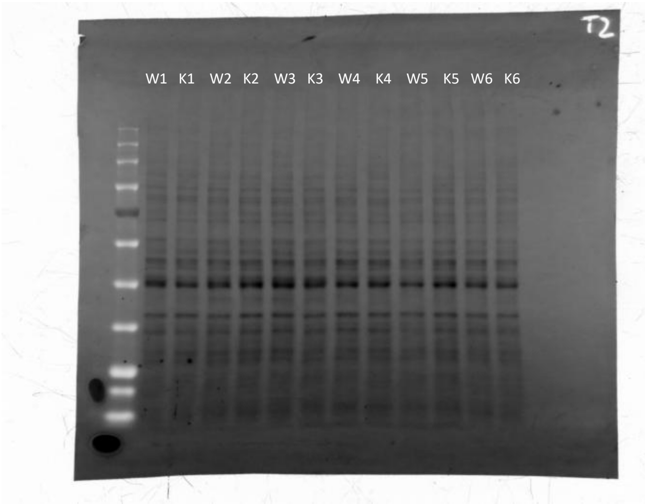

Western Blot – Figure 2F

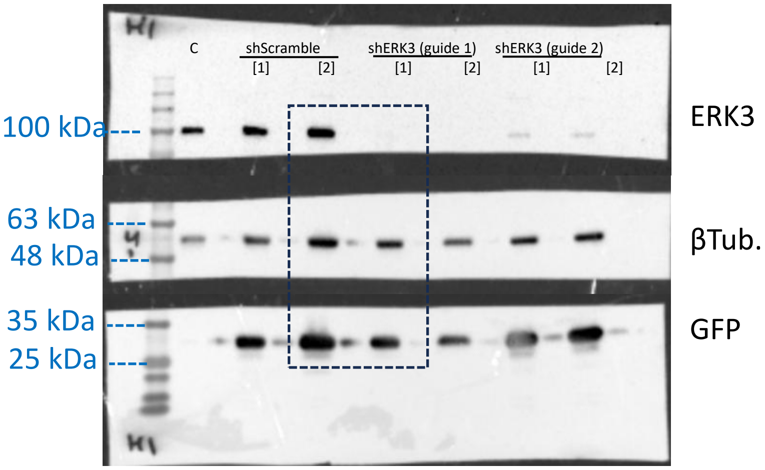

Corresponding Stain Free Blot

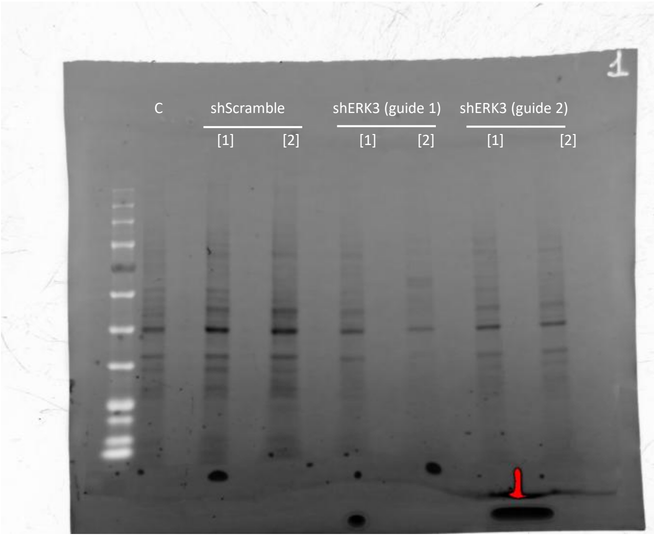

Western Blot – Figure 3A and Supplementary Figure 2G Corresponding Stain Free Blot

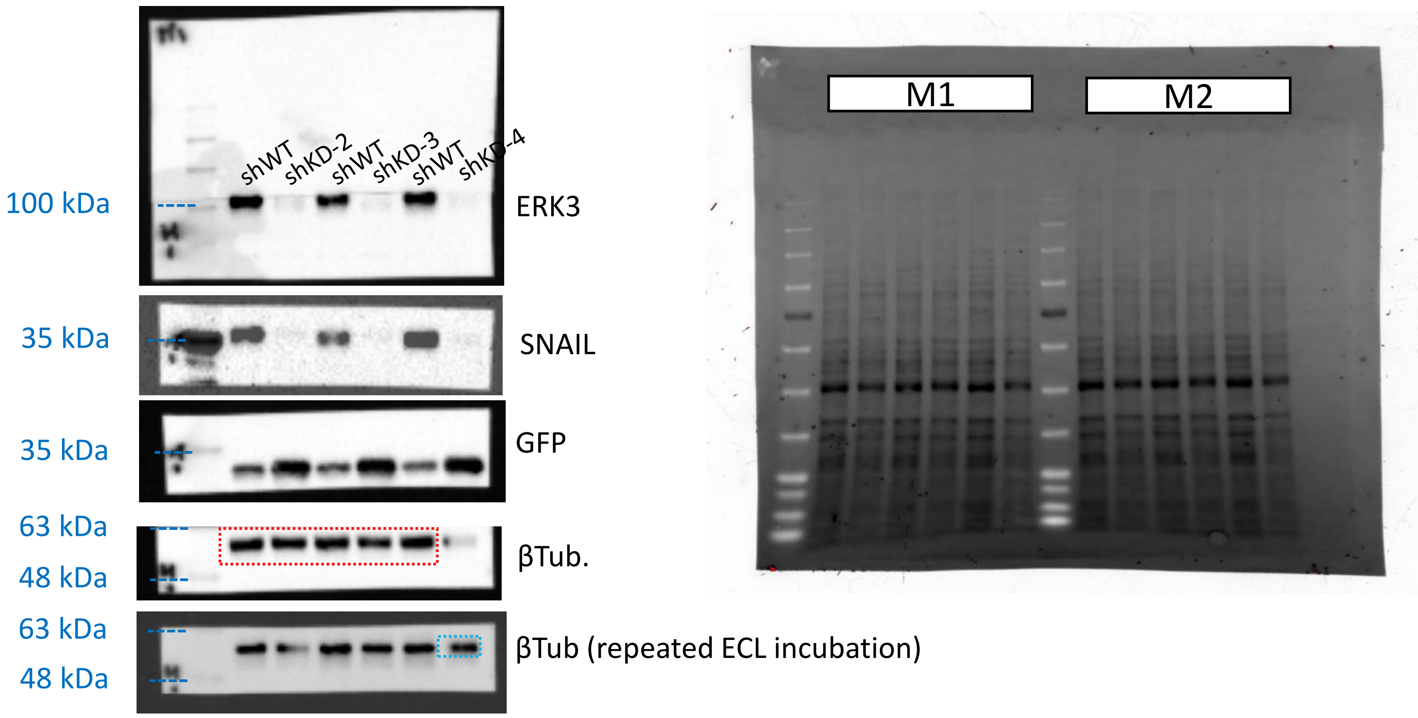

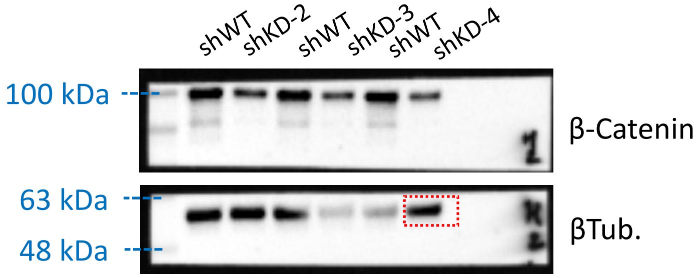

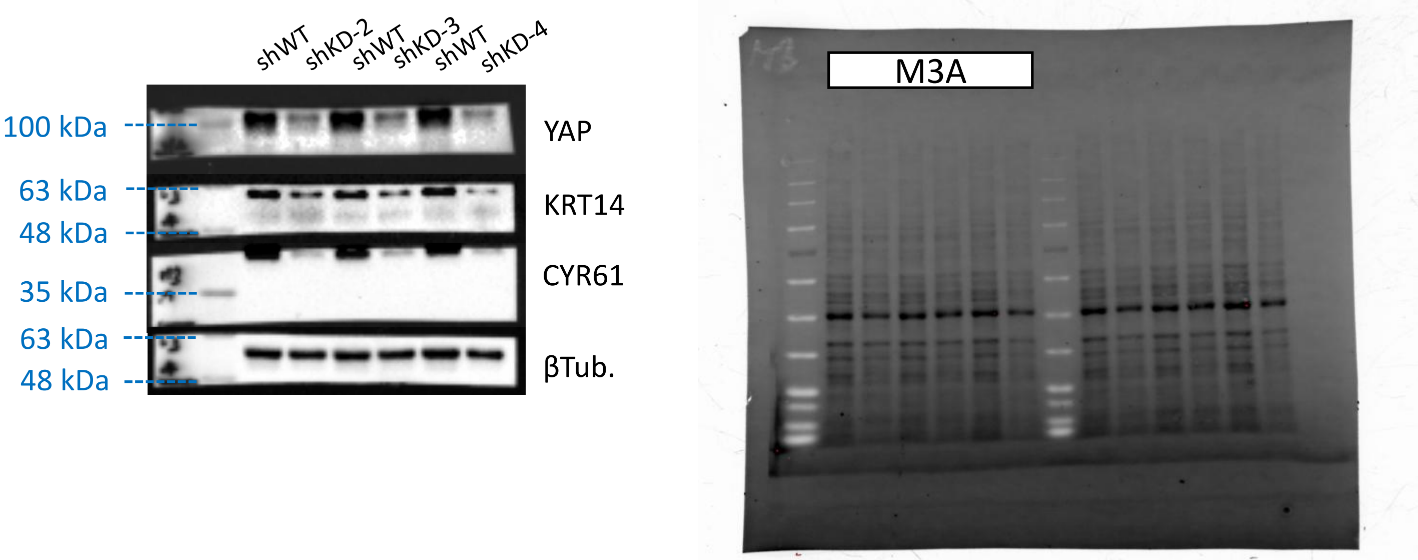

Western Blot – Supplementary Figure 2A

Corresponding Stain Free Blot

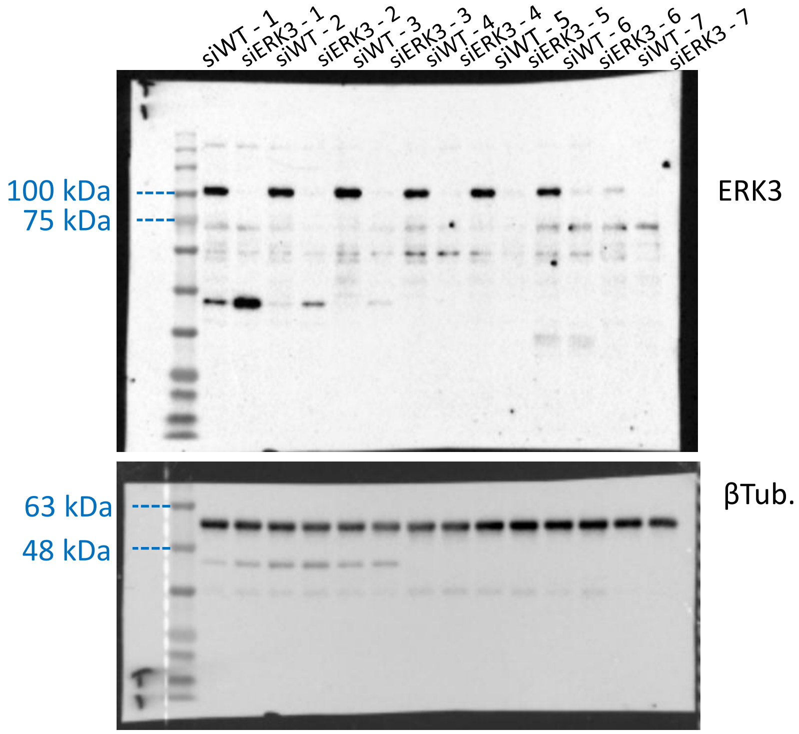

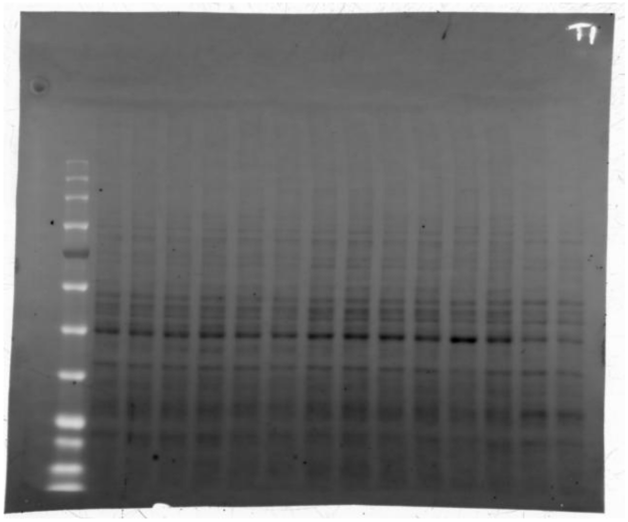

Western Blot – Supplementary Figure 2H

Corresponding Stain Free Blot

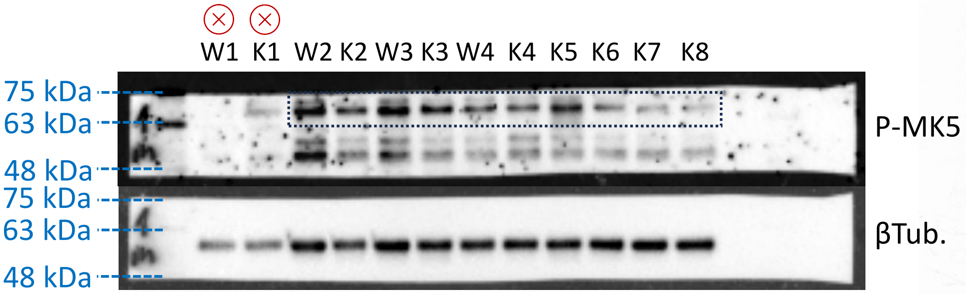

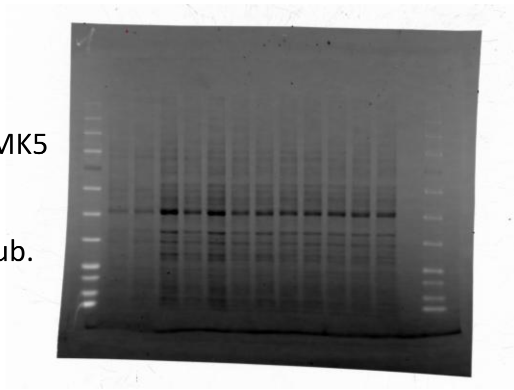

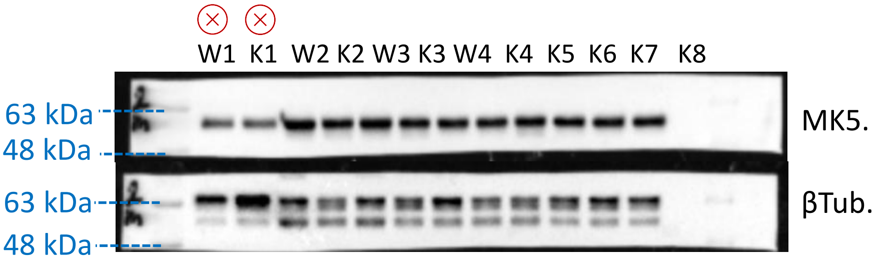

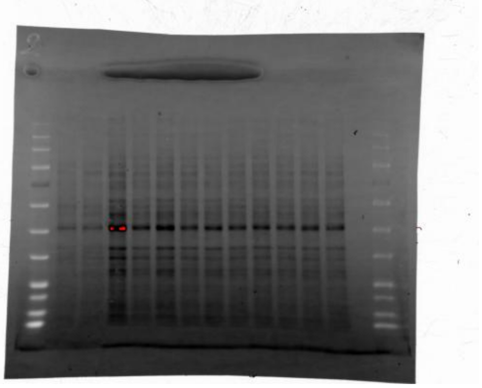

Western Blot – Supplementary Figure 2I

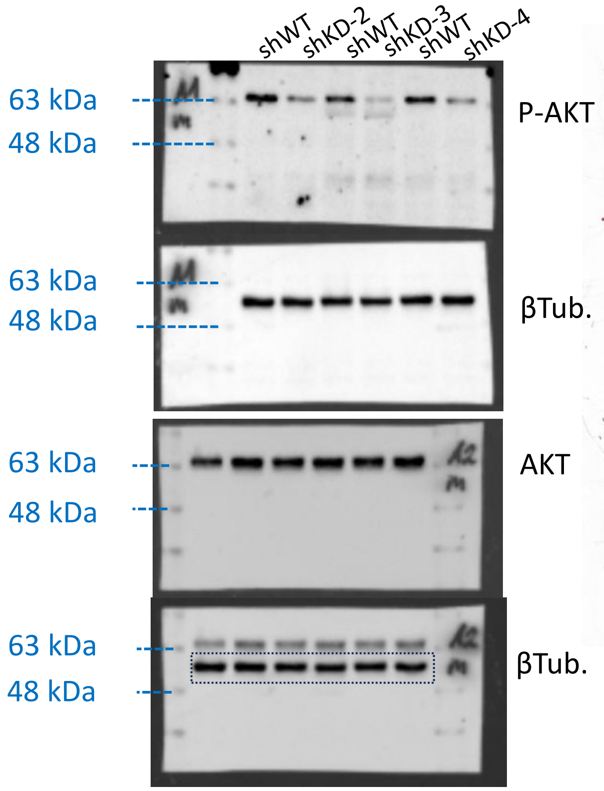

Corresponding Stain Free Blot

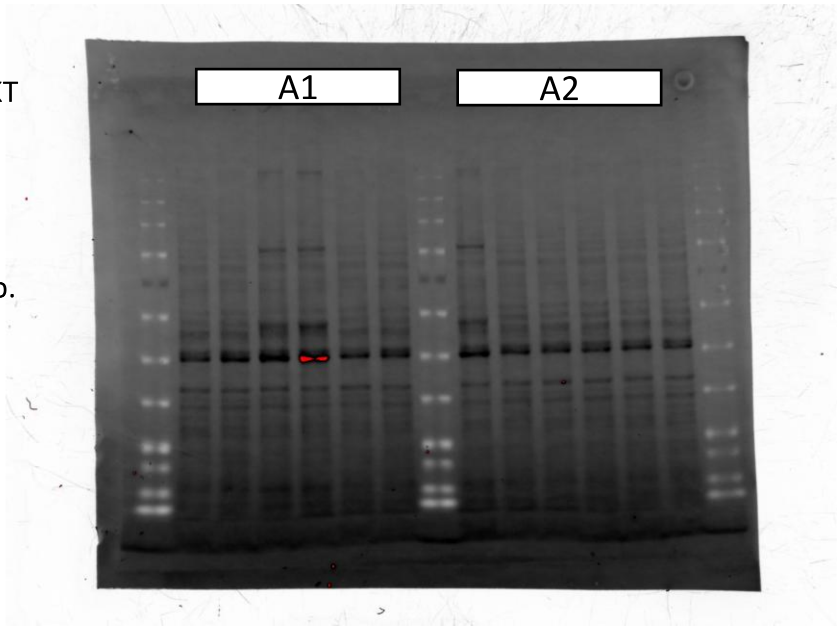

Western Blot – Supplementary Figure 3A

Corresponding Stain Free Blot

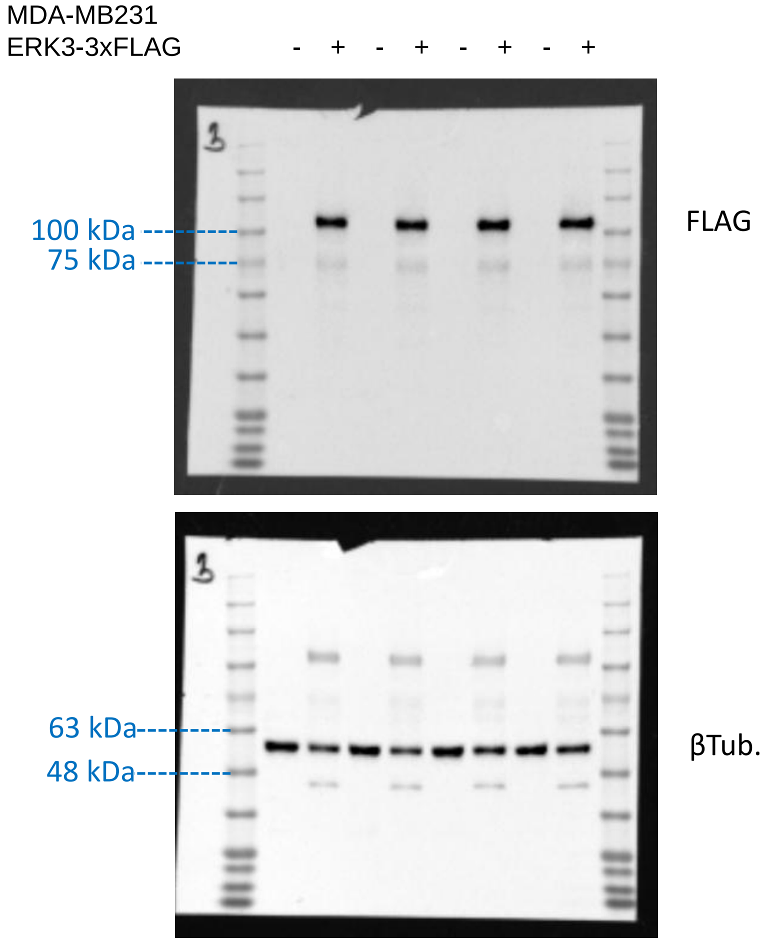

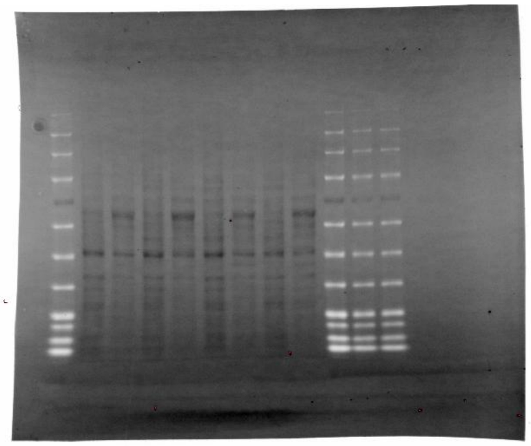

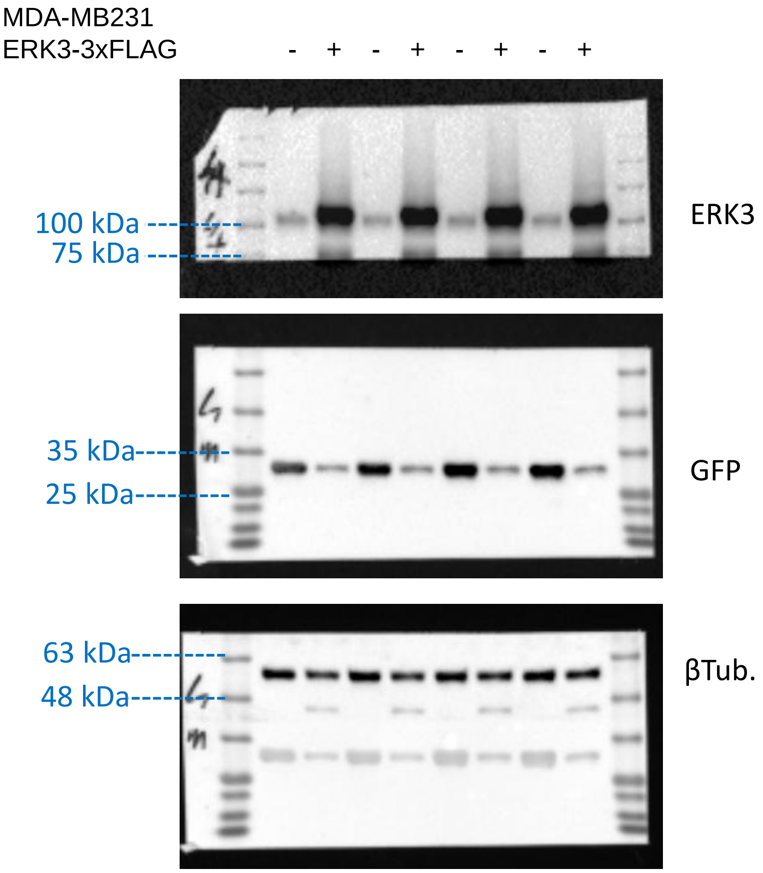

Corresponding Stain Free Blot

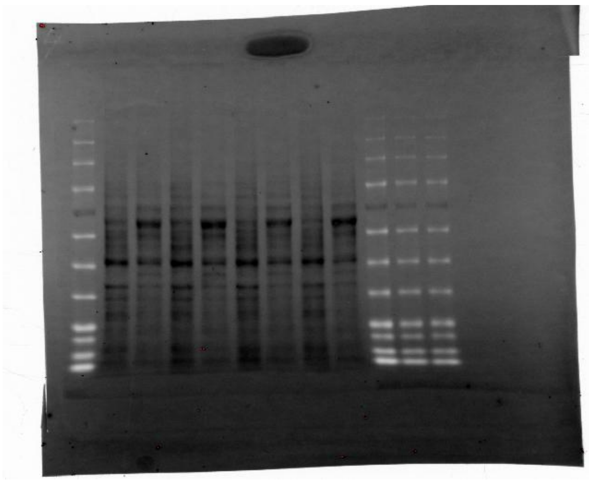

Western Blot – Supplementary Figure 4E

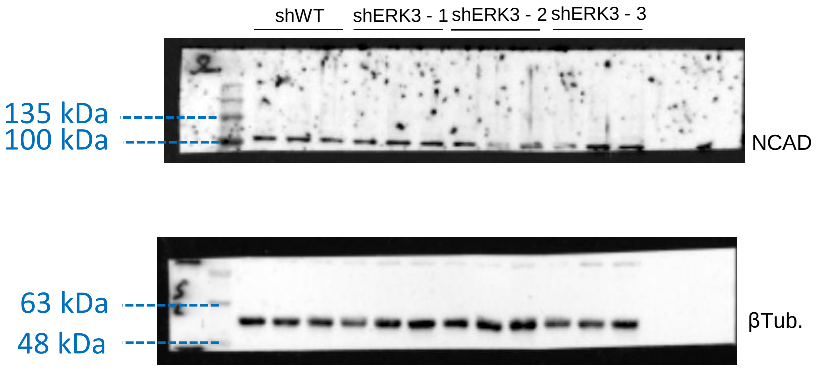

Corresponding Stain Free Blot

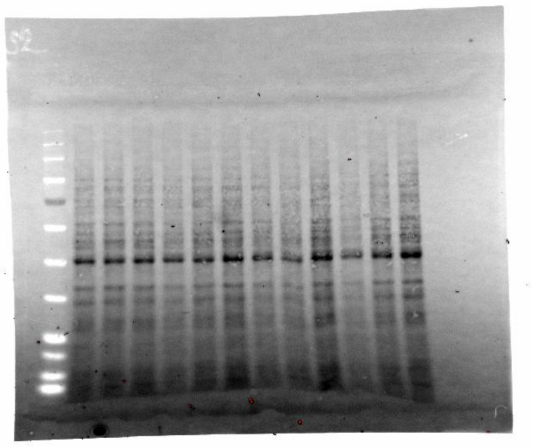

Western Blot – Supplementary Figure 5A

Corresponding Stain Free Blot

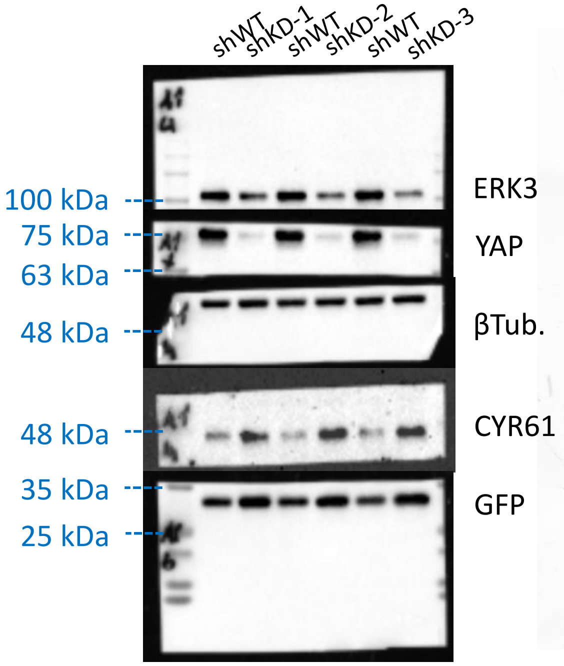

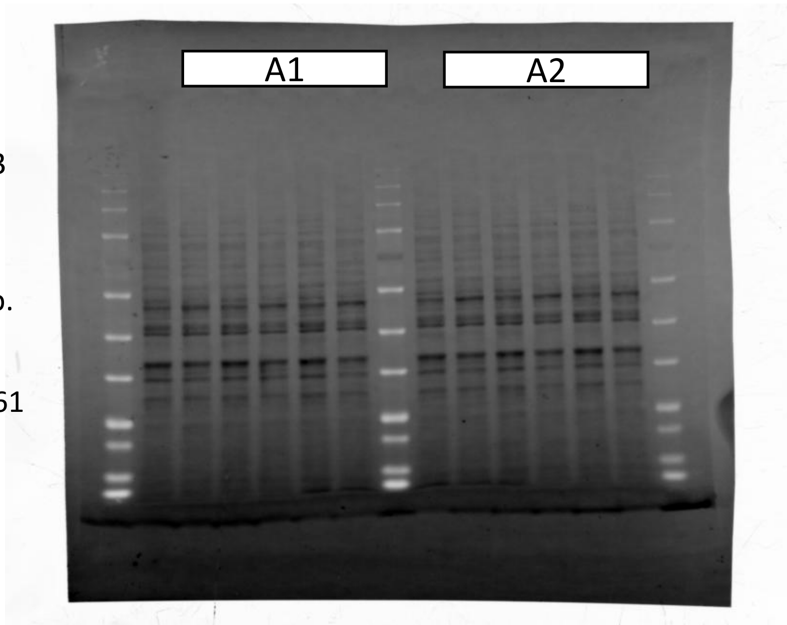

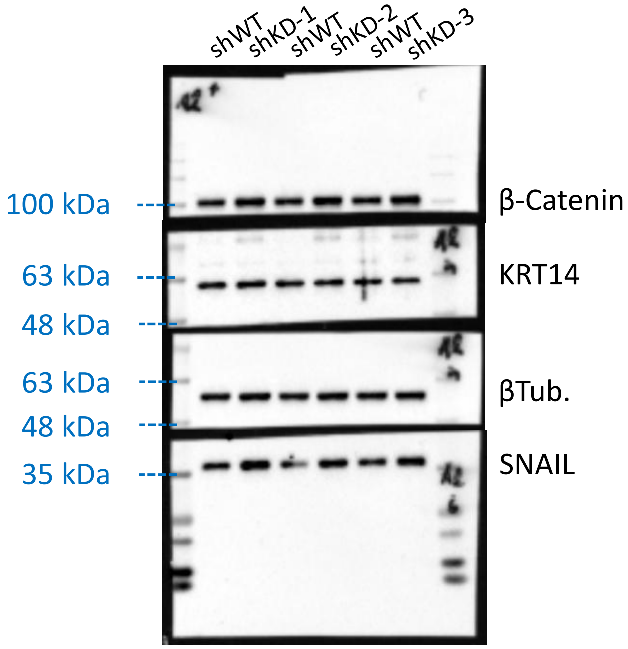

Western Blot – Supplementary Figure 6A

Corresponding Stain Free Blot

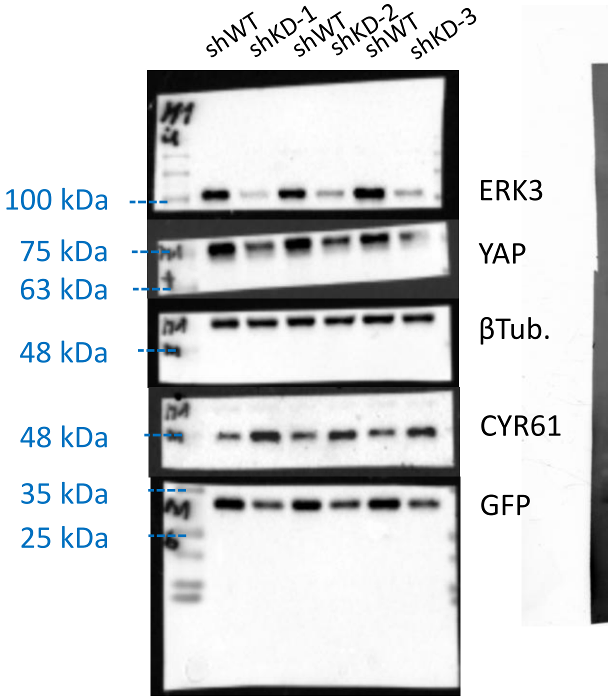

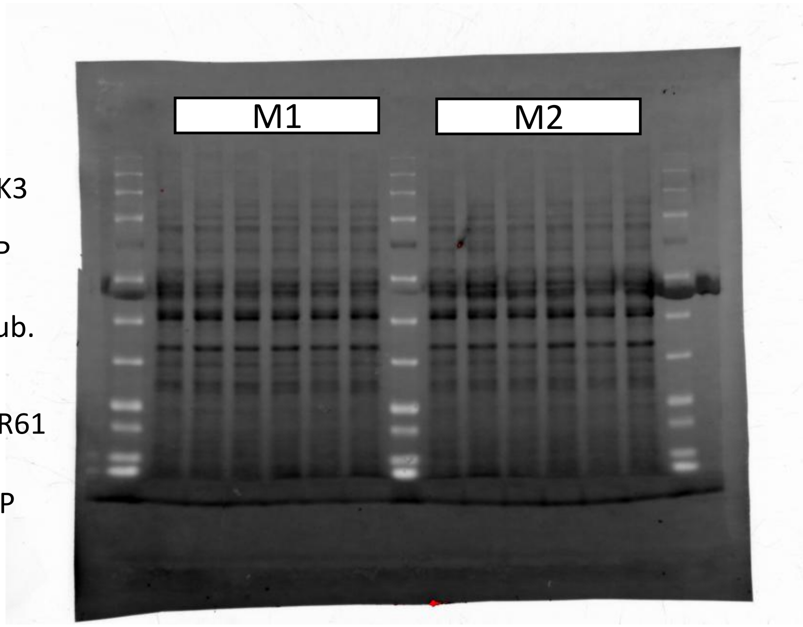

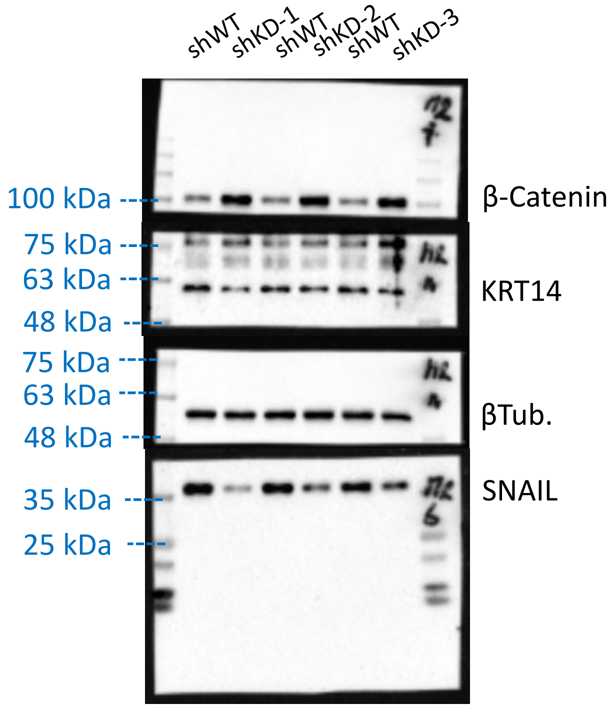

Supplement: Supplementary Datasheet 1 — Western Blots - Uncropped Western blot membranes and corresponding Stain-Free blot acquired images. [file DataSheet1.pdf]
